# Modulation of the Spin State of Atomic Fe-N_4_ Sites with Interlayer-Adjacent Ir-N_4_ for Superior ORR Activity

**DOI:** 10.1007/s40820-026-02108-9

**Published:** 2026-03-05

**Authors:** Yan Tan, Aoshuang Li, Yijie Wang, Xiucai Jiang, Yiwen Cheng, Dongliang Chao, Yuzhong Zhang, Chuanwei Cheng

**Affiliations:** 1 School of Physics Science and Engineering, Tongji University, Shanghai, 200092 People’s Republic of China; 2 Laboratory of Advanced Materials, Laboratory of Molecular Catalysis and Innovative Materials, Faculty of Chemistry and Materials, Aqueous Battery Center, Shanghai Key, Electron Microscope Center of Fudan University, Fudan University, Shanghai, 200433 People’s Republic of China

**Keywords:** Single-Atom catalysts, Spin-State transition, Interlayer interactions, 3D ordered microporous, Zn-Air batteries

## Abstract

Development of efficient and durable oxygen reduction reaction (ORR) electrocatalysts is of great interest yet remains challenging. Herein, we predicted and screened a bilayer graphite carbon-supported Ir-N_4_/Fe-N_4_ catalyst with high ORR activity using density functional theory calculations. Subsequently, various bimetallic single atom supported on 3D ordered macroporous carbon were rationally designed and experimentally synthesized via a colloidal microsphere template-confined reaction method. As anticipated, the resulting Ir-N_4_/Fe-N_4_ bimetallic single-atom catalysts (IrFe-SACs) exhibit superior ORR activity and durability, reaching a half-wave potential of 0.928 V. The IrFe-SACs also demonstrate outstanding performance in Zn-air batteries, including a high discharge power density (314 mW cm⁻^2^) and excellent cycling stability (~ 1650 cycles over 550 h). Further experimental characterizations and theoretical analysis reveal that introducing interlayer-adjacent Ir-N_4_ sites facilitates the transition of Fe-N_4_ from a low-spin state to a medium-spin state, which optimizes the spin polarization of Fe 3d orbitals and enhances the non-localization of the Fe–O/OH molecular orbital, thereby significantly improving the ORR intrinsic activity and durability of atomic Fe-N_4_ sites.

**Supplementary Information:**

The online version contains supplementary material available at 10.1007/s40820-026-02108-9.

## Highlights

- Ir-N_4_ induce spin polarization and trigger a low-spin to medium-spin transition at Fe centers, enhancing electron delocalization in Fe-O frontier orbitals, optimizes oxygen intermediate adsorption energies.
- Modulation of spin state of active center optimizes the adsorption energy and enhancing intrinsic catalytic activity (E_1/2_ = 0.928 V for oxygen reduction reaction).
- 3D ordered macroporous carbon framework ensures high accessibility of atomic sites, resulting in excellent batteries performance, i.e., a discharge power density of 314 mW cm^−2^, specific capacity of 817.5 mAh g^−1^.

**Supplementary Information:**

The online version contains supplementary material available at 10.1007/s40820-026-02108-9.

## Graphical abstract

**Figure:**
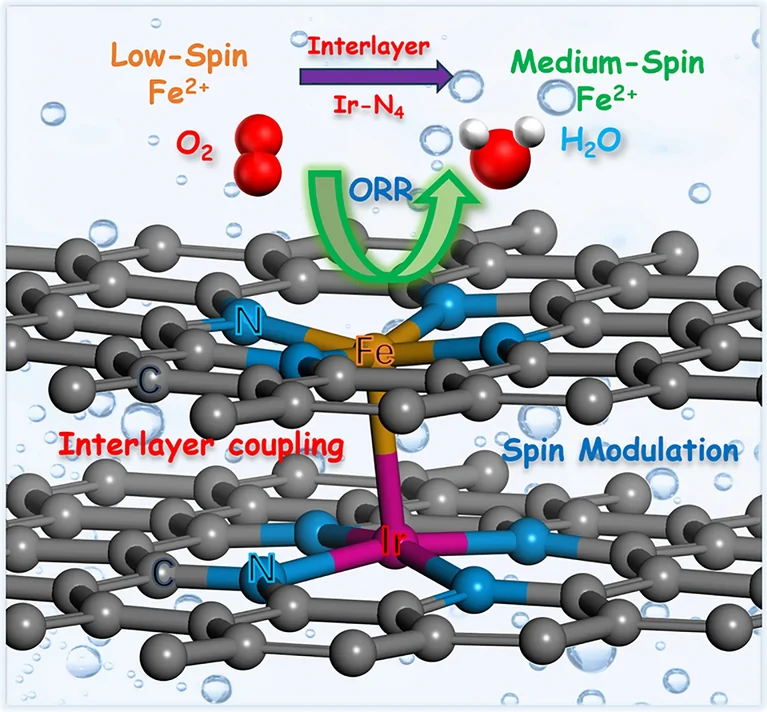

## Introduction

The oxygen reduction reaction (ORR) is critically important for metal-air batteries and fuel cells. However, sluggish multi-electron ORR kinetics still hinder the catalytic performance [1–3]. Developing efficient and durable ORR electrocatalysts is highly desirable yet challenging. Among reported electrocatalysts, carbon-supported single-atom catalysts (SACs) have received substantial interest due to their high intrinsic ORR catalytic activity and maximized use of active metal atoms [4–6]. The intrinsic ORR activity of carbon-supported SACs is closely correlated with the electronic structures and spin states of their metal active sites [7–10]. Strategies such as heterogeneous doping, defect engineering, and ligand environment modification can effectively adjust the electronic structure of atomic metal centers, induce spin-state transitions, and thereby enhance intrinsic ORR activity [11–13].

Despite significant progress in single-metal SACs, these catalysts remain inadequate for disrupting the linear correlation between the adsorption energies of multiple ORR reaction intermediates on single-metal active sites, which is a fundamental constraint on ORR activity [14–16]. In recent years, bimetallic SACs incorporating two distinct metal sites have emerged as a research hotspot in catalysis [17–19]. The synergistic interactions between dissimilar metals creates asymmetric adsorption sites that modify spin density and electronic structure of active centers, thereby optimizing intermediate adsorption energies and enhancing catalytic activity [20, 21]. To date, most investigated bimetallic SACs predominantly consist of monolayer configurations (M_1_-N_*x*_/M_2_-N_*x*_ or M_1_M_2_-N_*x*_, where M_1_, M_2_ represent different metals). However, such intralayer arrangements often exhibit unsatisfactory interactions between bimetallic sites, resulting in weak or excessive heteroatomic coupling and poor selectivity for oxygen intermediate adsorption [20–23]. While bilayer or multilayer carbon supported SACs naturally form during experiment synthesis, the interlayer interactions between vertically separated atomic metals are frequently overlooked [24–26]. Whereas during the synthesis process, bilayer/multilayer carbon structures are naturally formed. The interlayer interactions between vertically separated atomic metals are frequently overlooked. Moreover, the underlying mechanism of interlayer coupling on the catalytic property in bilayer bimetallic SACs was rarely explored. The incorporation of different metal sites into adjacent interlayers to create interlayer dual single-atom configurations induces pronounced spin coupling and electronic redistribution between neighboring sites. This interlayer spin modulation could effectively tune the adsorption energies of critical reaction intermediates, promote O–O bond cleavage and electron transfer, and thereby significantly enhance the ORR activity and kinetics. Moreover, the underlying mechanism of interlayer coupling on the catalytic property in bilayer bimetallic SACs remains unclear. Understanding how interlayer coupling modulates electronic structures and spin states of active sites in bimetallic SACs is extremely crucial for rationally designing high-performance ORR electrocatalysts.

Herein, we proposed a bilayer carbon-supported bimetallic single-atom model for predicting and screening high-performance ORR electrocatalysts through density function theory (DFT) calculations. The Ir-N_4_/Fe-N_4_ configuration was computationally identified as exhibiting a low theoretical overpotential and high ORR activity. Subsequently, various bimetallic SACs supported on 3D ordered macroporous graphitic carbon were rationally designed and experimentally synthesized via colloidal microsphere-templated ligand confinement reactions followed by a high-temperature pyrolysis process. As predicted, the bilayer Ir-N_4_/Fe-N_4_ catalyst demonstrated outstanding ORR activities with a half-wave potential of 0.928 V. More significantly, Zn-air batteries employing the Ir-N_4_/Fe-N_4_ catalyst achieved a peak power density of 314 mW cm⁻^2^ and exceptional cycling stability (∼1650 cycles over 550 h). In-situ Raman spectroscopy test and DFT analysis revealed Fe-N_4_ as the primary active site, where interlayer Ir-N_4_ induces a spin-state transition in Fe-N_4_ (from low-spin to medium-spin) via crystal field coupling. This spin polarization enhances electron non-localization in Fe–O frontier orbitals, optimizes oxygen intermediate adsorption energies, and reduces reaction energy barriers, thereby elevating the intrinsic ORR activity and durability of bilayer Ir-N_4_/Fe-N_4_ catalyst.

## Experiment Section

### Materials Synthesis

#### Preparation of 3D Ordered Macroporous (3DOM) Carbon Supported IrFe-SACs

First, 100 mL of 5 wt% polystyrene microsphere emulsion (microsphere diameter: ~ 300 nm) was taken in a centrifuge tube and centrifuged several times at 15,000 rpm, removed the supernatant and placed it in a 60 °C oven overnight to obtain monolithic polystyrene spheres template. Then, 5.07 g of 2-methylimidazole, 5.82 g of Zn(NO_3_)_2_·6H_2_O, and 0.505 g of iridium(III)2,4-pentanedionate were dissolved and dispersed in 15 mL of deionized water to form a precursor solution. The self-assembled polystyrene spheres template was put into the above precursor solution and impregnated at room temperature for 2 h. After that, the precursors infiltrated polystyrene template was placed in a solution of ammonia and methanol with a volume ratio 1:1 for 24 h. The impregnated template was filtered to obtain the Ir doped zeolitic imidazolate framework-8 (ZIF-8) filled template and dried in an oven at 50 °C overnight. The precursor-filled microsphere template was placed in the argon atmosphere of the tube furnace and calcined at 5 °C min^−1^ to 400 °C for 5 h, and then 5 °C min^−1^ to 920 °C for 3 h to burn out the polystyrene spheres and pyrolysis of Ir doped ZIF-8 to obtain 3DOM carbon supported Ir-SACs. Finally, 3DOM carbon supported IrFe-SACs was fabricated by coating another layer of Fe SACs doped carbon on Ir-SACs. 100 mg of 3DOM carbon supported Ir-SACs, 400 mg of FeSO_4_·7H_2_O, and 800 mg of o-phenanthroline were dispersed into 20 mL of ethanol and 10 mL of water. Sonication was performed at room temperature for 1 h and dried at 60 °C. The dried products were mixed with dicyandiamide at a mass ratio of 1:5 and calcined in a tube furnace under argon atmosphere at 5 °C min^−1^ for 2 h at 1000 °C to obtain IrFe-SACs. For comparison, 3DOM carbon supported CoFe-SACs, NiFe-SACs, CuFe-SACs, MnFe-SACs, RuFe-SACs were prepared with similar procedure except that iridium(III)2,4-pentanedionate was replaced by an equal number of moles of Co(NO_3_)_2_·6H_2_O, Ni(NO_3_)_2_·6H_2_O, Cu(NO_3_)_2_·3H_2_O, Mn(NO_3_)_2_·4H_2_O, and RuCl_3_·*x*H_2_O, respectively. 3DOM carbon supported Ir-SACs, Fe-SACs and IrFe-SACs without 3DOM structures were prepared without the addition of FeSO_4_·7H_2_O, iridium(III)2,4-pentanedionate and polystyrene spheres template in the corresponding synthetic process, respectively. IrFe-SACs (one-step) were prepared by adding all the metal salts and organic ligands involved in the above two-step method together when preparing the precursor solution for the first time, drying and then grinding with five times the mass of dicyandiamide, followed by pyrolysis at 1000 °C in an argon atmosphere for 2 h.

### Electrochemical Performance Tests

Electrocatalytic performance was evaluated using a three-electrode system with the as-prepared samples as the working electrode, a Hg/HgO electrode (1.0 M KOH) as the reference electrode, and a graphite rod as the counter electrode. Under ultrasonic treatment, 5 mg of as-prepared catalysts were completely disseminated in 50 µL of 5% Nafion, 150 µL of ethanol, and 300 µL of deionized water. All samples received the same mass loading, and were dried naturally before the test. All measurements were carried out on a CHI760D electrochemical workstation (Chenhua, Shanghai, China). Linear sweep voltammetry (LSV) tests for ORR were performed in O_2_-saturated 0.1 M KOH at a scan rate of 5 mV s⁻^1^ using a rotating disk electrode (1600 rpm). Commercial of Pt/C and RuO_2_ were used for comparison. The Zn-air batteries performance was evaluated by assembling aqueous Zn-air batteries with a pre-polished zinc foil as anode, an electrolyte consisting of 6.0 M KOH with 0.2 M Zn(CH_3_COO)_2_, and an air cathode composed of the sample, nickel foam, and a hydrophobic layer, with an exposed surface area of 1 cm^2^. Polarization curves for charge and discharge were captured using a CHI 760D electrochemical workstation. Cycling tests were performed on a Neware Test System CT-3008 at a current density of 5 mA cm⁻^2^, with alternating 10-min discharge and 10-min charge cycles. The specific capacity of the ZABs was determined based on the consumption of zinc on the anode.

### Characterizations

The structure and morphology of the samples were observed by field emission scanning electron microscope (FE-SEM, FEI sirion200), high-resolution transmission electron microscope (HR-TEM, FEI-Talos F200X) and high-angle annular dark-field scanning transmission electron microscope (HAADF-STEM, FEI Titan G2 60–300, 300 kV). The X-ray absorption fine structure (XAFS) spectra data were taken at 1W1B station of the Beijing Synchrotron Radiation Facility (BSRF, operated at 2.5 GeV). Inductively coupled plasma mass spectroscopy (ICP-MS, Agilent 7700) was used to determine the contents of Ir and Fe. Electron paramagnetic resonance (EPR) spectra were collected by EPR spectroscopy (Bruker-A300). The temperature-dependent magnetic susceptibility (M-T) in zero-field-cooling (ZFC) was measured with a physical property measurement system (Quantum Design PPMS-9). The surface composition and valence states were recorded using X-ray photoelectron spectroscopy (XPS, Thermo ESCALAB250XI). In-situ Raman spectroscopy was measured by DEEP-INRS-II at 200 ~ 1800 nm laser excitation, employing IrFe-SACs based electrode as the working electrode, a platinum wire as the counter electrode and Hg/HgO electrode serves as the reference electrode, and 0.1 M KOH was used as electrolyte.

### Theoretical Calculation

Based on density function theory (DFT), the projector-augmented wave approach as implemented in the Vienna Ab-initio Simulation Package (VASP), was used to calculate reaction free energy differences of IrFe-SACs, CoFe-SACs, NiFe-SACs, PtFe-SACs, RuFe-SACs, MnFe-SACs, ZnFe-SACs, CuFe-SACs, Ir-SACs, and Fe-SACs. The exchange–correlation potential was computed within the spin-polarized generalized gradient approximation (GGA) of Perdew-Burke-Ernzerhof (PBE), and all atoms were relaxed. We employed DS-PAW(a computational software from HZWTECH [27]) for frequency calculations and free energy corrections to ascertain the contributions of zero-point vibrations and entropy to Gibbs free energy. More calculation details can be found in Supporting Information.

## Results and Discussion

### Model Establishment and DFT Screening

**Fig. 1:**
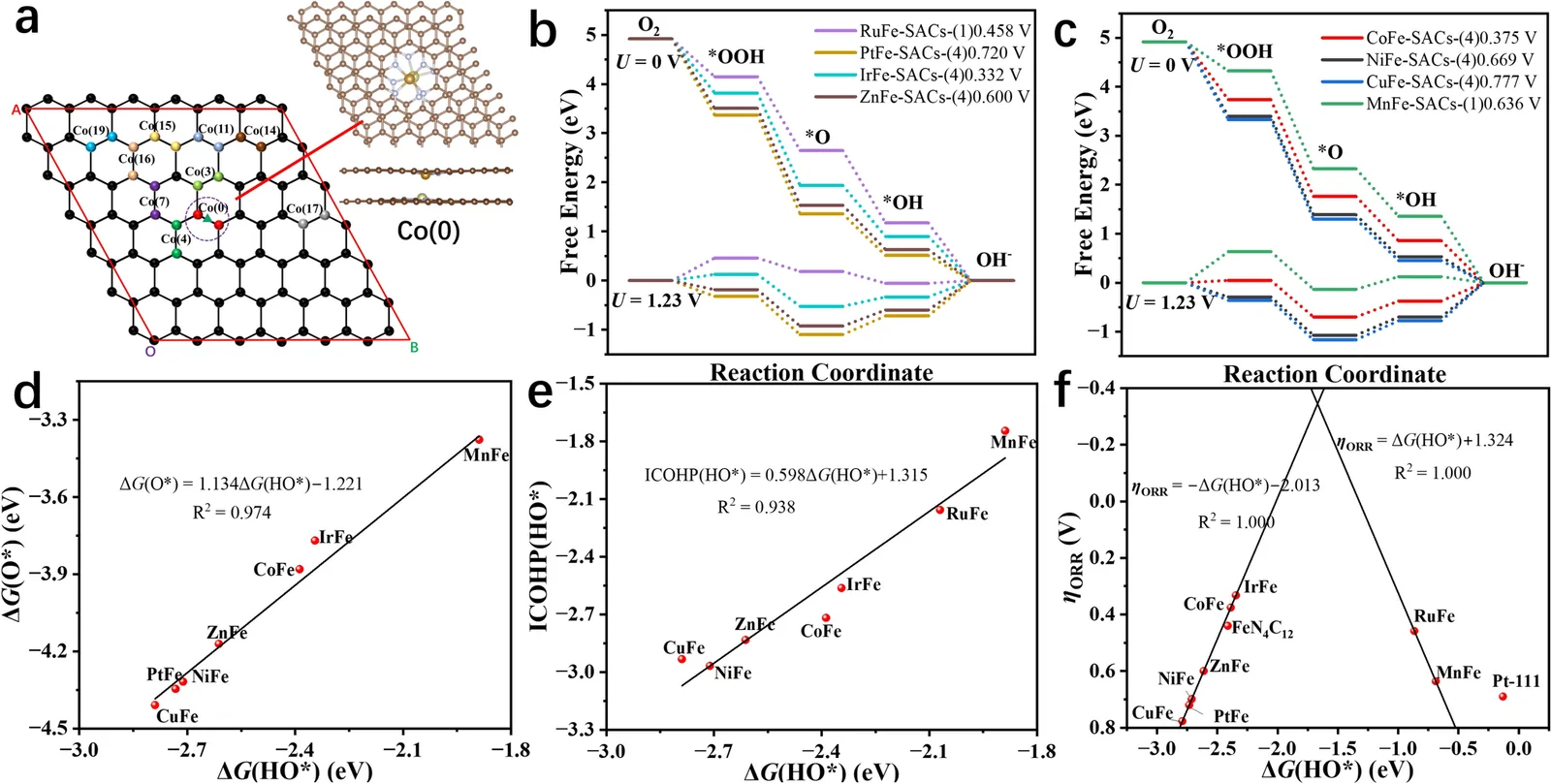
**a** Possible bilayer bimetallic atomic catalyst configurations. **b, c** ORR path free energy step diagrams for different bimetallic atomic configurations. Conformational relationships between Δ*G*(*OH) and **d** Δ*G*(*O), **e** ICOHP(*OH), **f** ORR theoretical overpotentials

Theoretical evaluation of optimal atomic configurations by DFT simulations not only accelerates the synthesis of highly active single-atom catalysts but also provides mechanistic insights for guiding experimental optimization. For bimetallic systems, both the spatial arrangement and elemental composition of bimetallic atom sites critically determine the intrinsic ORR activity [28, 29]. In this work, we systematically constructed 6 × 6 bilayer graphene supercells containing coordinated M-N_4_ moieties with various transition metal combinations. The structural stability of these bimetallic catalysts was found to be extremely reliant on the relative position and geometric orientation of the metal centers within the bilayer carbon architecture. As a representative case study, we employed a CoFe bimetallic single-atom configuration with Fe-N_4_ sites anchored on the top graphene layer and Co-N_4_ sites positioned on the bottom layer. By fixing the Fe-N_4_ coordination geometry while systematically varying the relative positions of Co-N_4_ moieties, we successfully constructed 20 distinct CoFe single atomic catalyst models (Figs. 1a and S1). Utilizing extensive structural relaxation and self-consistent calculations, we employed DS-PAW to screen and acquire the best bimetallic spatial configuration. The ground-state energy (*E*) and intermetallic distances (*d*) for all configurations are presented in Table S1. As a result, the most stable bimetallic atomic configuration is found to be Co(0) (*d* = 2.511 Å, *E* = − 1309.097 eV), which demonstrates that the CoFe-SACs with the shortest interlayer atomic metal spacing would exhibit the strongest interlayer interactions. The atomic configuration of the reference Co(0) site is illustrated in the inset of Fig. 1a.

Building upon the optimized bilayer bimetallic configurations, we systematically evaluated the ORR catalytic performance of various transition metal combinations. In comparative study, the top-layer Fe single-atom site was maintained as a constant while systematically substituting the bottom-layer metal with Co, Ni, Cu, Mn, Zn, Ir, Ru, and Pt, thereby generating eight distinct bimetallic single-atom architectures. For each configuration, we calculated the complete ORR reaction step diagram. As evidenced by Fig. 1b, c and Table S2, the IrFe-SACs demonstrates exceptional catalytic performance, exhibiting the lowest theoretical overpotential of 0.332 V among all investigated combinations, which indicates that the IrFe bimetallic configuration possesses superior intrinsic ORR activity. As shown in Figs. S2, S3 and Table S3, compared with the interlayer configuration, such intralayer Ir–Fe dual-atom structures and isolated Ir or Fe single atoms display significantly lower ORR activity and are unlikely to account for the high catalytic performance observed experimentally. Furthermore, results in Table S3 and Figs. S2, S3 indicate that carbon defects have a modest effect on the ORR activity. To quantitatively assess the binding strength of oxygen intermediates on the catalyst surface during the ORR process, we conducted a comprehensive analysis of the crystal orbital *Hamiltonian* population (COHP) for metal–oxygen bonds (Table S4). The calculation results reveal that IrFe-SACs in rate-limiting step exhibits an optimal integral COHP (ICOHP) value of -2.56 for the Fe-OH bond, representing the most favorable electronic interaction strength among all investigated catalysts. This intermediate binding strength enables balanced adsorption/desorption kinetics for both oxygen intermediates and reaction products, which is crucial for improving high ORR activity. Furthermore, we observed a significant linear correlation among the three key parameters: ICOHP(*OH), Δ*G*(*O), and Δ*G*(*OH), suggesting some descriptors for predicting ORR catalytic performance, i.e., ICOHP(*OH) = 0.598Δ*G*(*OH) + 1.315, *R*^2^ = 0.938; Δ*G*(*O) = 1.134Δ*G*(*OH)−1.221, *R*^2^ = 0.974, as plotted in Fig. 1d, e. Furthermore, the correlation between theoretical ORR overpotentials and Δ*G*(*OH) for various bilayer bimetallic SACs exhibits a characteristic volcano-shaped relationship, as illustrated in Fig. 1f. Notably, the bilayer IrFe-SACs configuration occupies favorable position near the apex of this volcano plot, suggesting that the bilayer IrFe-SACs system possesses exceptional intrinsic ORR catalytic activity.

### Materials Preparation and Characterizations

**Fig. 2:**
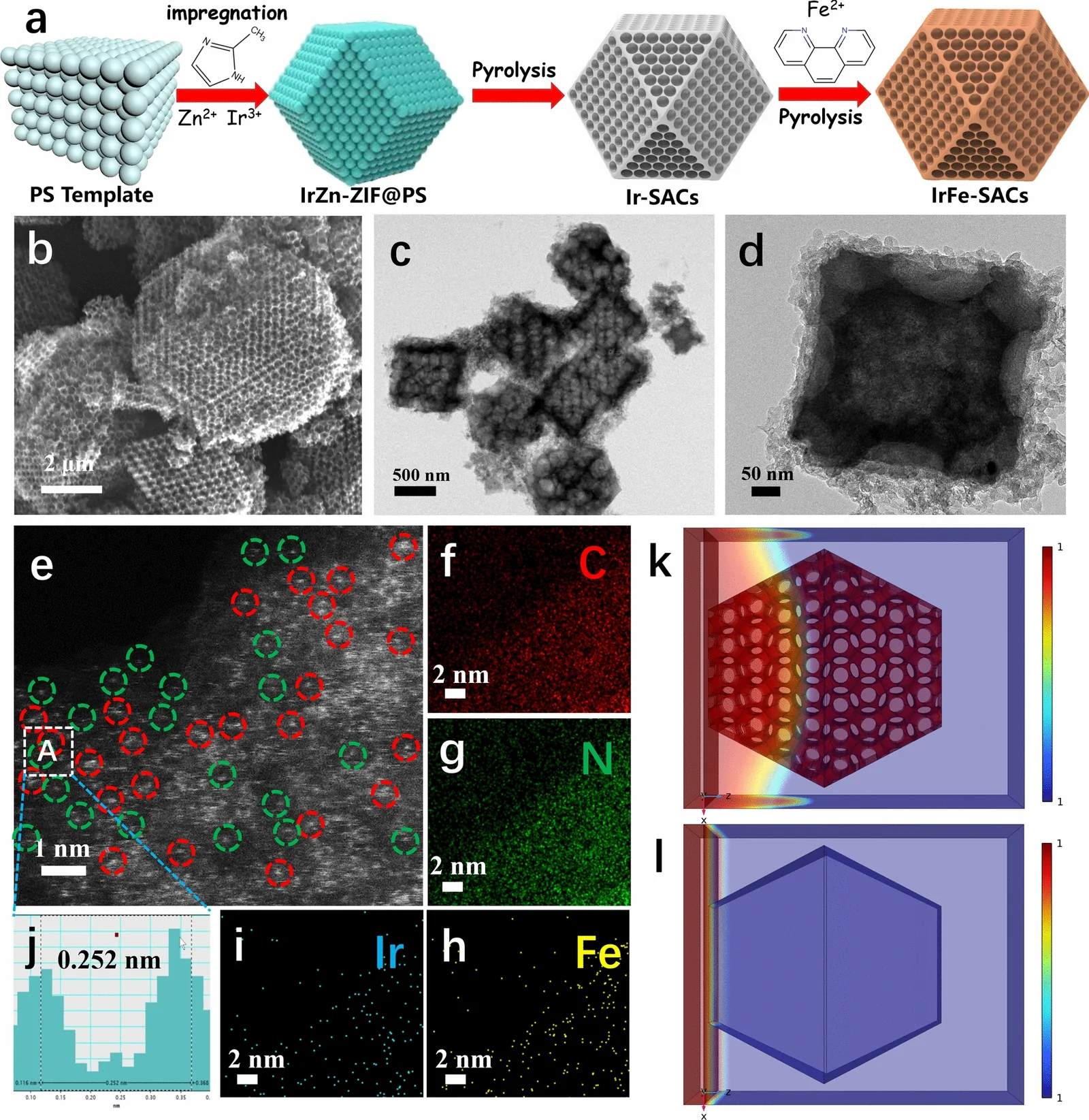
**a** Schematic illustration of the preparation, **b** SEM images, **c, d** HR-TEM images, and **e** HAADF-STEM images, **f-i** element mapping and **j** the intensity profiles in the A region of IrFe-SACs. Multi-physics field simulations of O_2_ diffusion and concentration distribution on **k** 3D ordered macroporous and **l** non-porous carbon surfaces

Inspired by the findings of the aforementioned DFT calculations, we rationally designed and successfully synthesized a series of 3DOM carbon supported bilayer bimetallic SACs through a colloidal microsphere template-confined synthesis strategy coupled with controlled pyrolysis. Figure 2a illustrates typical preparation process for IrFe-SACs. First, 3DOM carbon loaded Ir-SACs was obtained by impregnation of 2-methylimidazole, iridium (III)2,4-pentanedionate and zinc nitrate precursors solution in the voids of monolithic polystyrene microsphere templates to form a Ir doped ZIF-8 precursor by combination of a subsequent high-temperature pyrolysis step to stabilize the Ir-SACs and remove the polystyrene sphere template. Subsequently, a secondary carbon layer decorated with Fe single-atom sites was deposited onto the pre-formed 3DOM carbon supported Ir-SACs substrate, thereby constructing a well-defined bilayer architecture. This bilayer structure features an inner macroporous carbon layer stabilized with isolated Ir atoms and an outer carbon matrix uniformly anchored with Fe single-atoms. Following this established protocol, we successfully extended the synthesis to a series of bimetallic SACs including NiFe, CoFe, MnFe, RuFe, and CuFe systems by varying the metal type of the inner ZIF. Structural characterization reveals that the as-prepared IrFe-SACs maintain a highly ordered 3D macroporous framework with uniform pore diameter distribution centered at ~ 200 nm (Fig. 2b), while representative SEM images of other bimetallic analogues are provided in Fig. S4. The interconnected porous architecture is further corroborated by TEM analysis (Figs. 2c, d, S5 and Table S5). Higher atomic number elements possess a stronger ability to scatter electrons, thus appearing brighter in ADF-STEM images. As shown in Fig. 2e-i, numerous atomic-scale bright spots are observed on the sample surface, among which some appear significantly brighter. These correspond to signals from heavier atoms, whereas the dimmer spots represent lighter atoms. Given that Ir and Fe are the only possible metal single atoms in this sample, it is reasonable to attribute the bright spots to Ir single atoms and the dimmer ones to Fe single atoms. And the intensity profiles in the marked a region reveals a 0.252 nm distance between Ir and Fe atoms, which agrees with the calculation results (Fig. 2j). The ICP analysis of Ir and Fe composition is shown in Table S6. The carbon-supported Ir single atoms synthesized in the first step were obtained through the pyrolysis of MOF derivatives, which led to the Ir atoms being predominantly embedded deep within the carbon layers derived from the pyrolysis of ZIF-8. Subsequently, Fe single atoms were introduced via adsorption onto the surface of these carbon supports. Consequently, the Ir and Fe single atoms are expected to mainly exhibit an interlayer spatial configuration. Notably, the rationally designed 3DOM carbon architecture offers two critical advantages for ORR: abundant exposed active sites and significantly enhanced mass transport properties. Futhermore, The majority of XRD peaks in Fig. S6 belong to the C (0 0 2)/(0 1 2)/(0 1 3) of carbon substrate, and there are no obvious signal peaks belonging to Ir or Fe nanoparticles, indicates that Ir/Fe could be highly atomically dispersed on the carbon matrix. Multi-physics simulations combined with surface wettability measurements (Figs. 2k, l and S7) demonstrate that superhydrophobic 3DOM structure establishes a steeper oxygen concentration gradient at the electrode–electrolyte interface compared to the non-porous counterparts. The 3D interconnected pore channels would facilitate efficient oxygen diffusion pathways, while the optimized surface wettability promotes effective triple-phase (gas–liquid-solid) boundary formation [30].

**Fig. 3:**
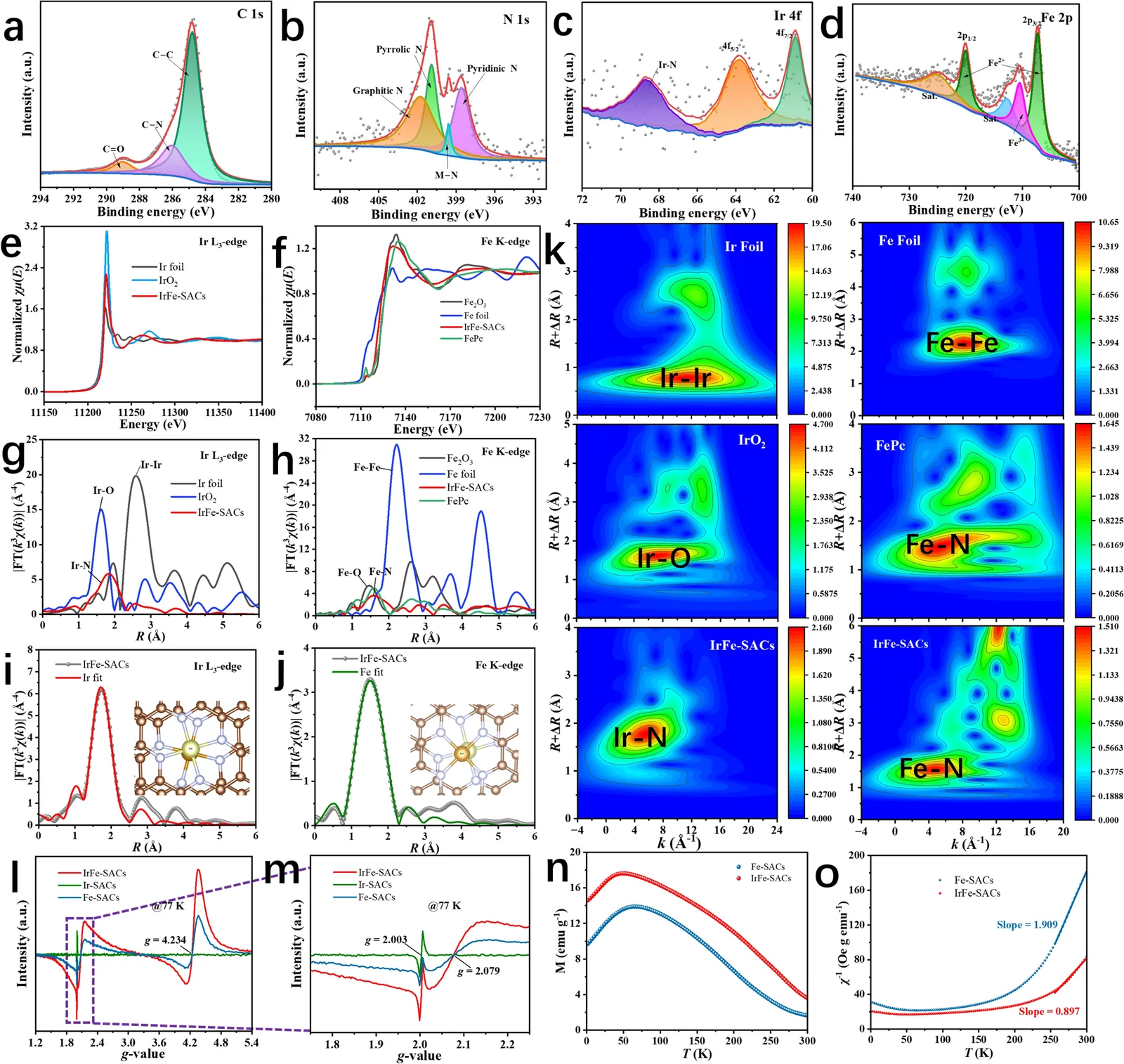
High-resolution XPS fine spectra of **a** C 1*s*, **b** N 1*s*, **c** Ir 4*f*, **d** Fe 2*p* for IrFe-SACs. **e, f** Normalized XANES spectra and **g, h** *Fourier* transforms of EXAFS spectra at the Ir L_3_-edge and Fe K-edge of IrFe-SACs and the reference materials at *R* space. XAFS curves represent fitted data based on **i** Ir L_3_-edge and** j** Fe K-edge experimental data, with the fitted curves of IrFe-SACs catalyst at the insets. **k** Wavelet transform of Ir L_3_-edge and Fe K-edge EXAFS spectra for Ir foil, Fe foil, IrO_2_, FePc, and IrFe-SACs, respectively. **l** EPR spectroscopy and **m** corresponding enlarged view for IrFe-SACs, Ir-SACs and Fe-SACs.** n** *M*-*T* plots and **o** temperature dependence inverse susceptibilities for IrFe-SACs and Fe-SACs

X-ray photoelectron spectroscopy(XPS) and X-ray absorption fine structure (XAFS) spectroscopy were used to systematically analyze the chemical composition and electronic states of IrFe-SACs. The high-resolution C 1*s* spectra in Fig. 3a can be decomposed into three peaks of C–C (284.8 eV), C–N (286.3 eV), and C=O (289.0 eV). The high-resolution N 1*s* XPS spectra in Fig. 3b may be fitted to four characteristic peaks of pyridine N (398.57 eV), pyrrole N (400.9 eV), graphite N (401.7 eV) and M–N (399.6 eV). The existence of M–N in IrFe-SACs suggests that Ir and Fe single-atoms are existing in Ir-N_x_ and Fe-N_x_ species, respectively [31]. The iridium species exhibiting distinctive peaks at 61.0 and 63.9 eV in the high-resolution XPS spectra of the Ir 4*f* area (Fig. 3c) are linked to Ir^4+^ [32]. The Ir 4*f* spectrum displays a weak signal, likely due to the encapsulation of most Ir atoms within the Fe–N–C shell layer. The Fe 2*p* spectrum in Fig. 3d can be deconstructed into Fe^2+^ (707.3 eV, 720.0 eV) and Fe^3+^ (710.4 eV) peaks, indicating the possible presence of Fe-N_x_ coordination bonds and more iron elements exists in the form of Fe^2+^. The electronic structure and local environment of Ir/Fe atoms in IrFe-SACs were further verified using XAFS. Figure 3e, f shows the Ir L_3_-edge and Fe K-edge X-ray absorption near edge structure (XANES) of IrFe-SACs and reference materials, respectively. The Ir L_3_-edge absorption of IrFe-SACs located between Ir foil and IrO_2_, indicating that the Ir in IrFe-SACs is in oxidized states ranging from 0 to + 4. The Fe K-edge absorption of IrFe-SACs located between Fe foil and Fe_2_O_3_, and similar to FePc, indicating that the Fe in IrFe-SACs is in oxidized states ranging from 0 to + 3 and close to FePc [33]. Fourier transform *k*^3^-weighted extended X-ray absorption fine structure (EXAFS) spectra in Fig. 3g, h shows that IrFe-SACs were rationalized and attributed to the coordination of Ir-N and Fe–N bands centered at ~ 1.8 and ~ 1.5 Å, suggesting that the Ir/Fe atoms are surrounded by N atoms and anchored to the carbon matrix [34, 35]. As shown in Figs. 3i, j, S8 and Table S7, according to the EXAFS fits, the IrN_4_/FeN_4_ configuration is present in the core Ir/Fe site of IrFe-SACs, as evidenced by the first Ir–N/Fe–N scattering routes of IrFe-SACs having a coordination number of four. And Ir-Fe scattering paths with a coordination number of one demonstrate the strong interaction between neighboring atomic Ir/Fe. The wavelet transform (WT) EXAFS is a powerful technique for making elemental distinctions between backscattered atoms. Figure 3k illustrates that WT contour maps of IrFe-SACs show a single maximum intensity value in *k*-space at around 6.0/4.5 Å^−1^, attributed to Ir-N/Fe–N species, distinguishing it from WT plots of Ir/Fe foil and IrO_2_, similar to FePc, confirming the presence of atomic Ir/Fe in IrFe-SACs [14, 32]. Additionally, XAFS results in Fig. S9 and Table S8 indicate the dominant scattering paths correspond to Fe–N coordination for Fe-SACs. Electron paramagnetic resonance (EPR) spectroscopy reveals pronounced electronic modulation induced by the incorporation of Ir–N_4_ moieties. As shown in Fig. 3l, Ir-SACs display only a weak signal near *g* ≈ 2.003, which can be attributed to carbon-related defects. Compared to Ir-SACs, both IrFe-SACs and Fe-SACs exhibit a distinct resonance signals centered at *g* ≈ 2.079 and *g* ≈ 4.234, which should be characteristic of the magnetism for atomic Fe species (Fig. 3m) [36–38]. Notably, the EPR signal intensity of IrFe-SACs is significantly higher than that of Fe-SACs, indicating that the introduction of atomically dispersed Ir effectively enhances the magnetic response of Fe sites. By comparison, Ir-SACs remain essentially nonmagnetic, in good agreement with the DFT-calculated results. Temperature-dependent magnetization measurements were carried out under a magnetic field of 300 Oe using zero-field-cooling (ZFC) procedures for both IrFe-SACs and Fe-SACs (Fig. 3n, o). The high-temperature area (T ≥ 250 K) was matched with the relevant magnetic susceptibilities obtained from the ZFC data. based on Curie–Weiss law, yielding Curie constants of 1.115 emu K g^−1^ Oe^−1^ for IrFe-SACs and 0.524 emu K g^−1^ Oe^−1^ for Fe-SACs. The number of unpaired *d* electrons (*n*) at the Fe and Ir sites was estimated based on both the M–T measurements and the equation $2.828\sqrt{{\chi }_{m}T}={\mu }_{eff}=\sqrt{n(n+2)} {\mu }_{B}$ [36]. The number of unpaired *d* electrons at the Fe site was calculated to be ~ 0.1 for Fe-SACs (*μ*_eff_ = 0.46 *μ*_B_), indicating that atomic Fe mainly adopts a low-spin (LS) configuration with mixed valence states. In contrast, the total number of unpaired *d* electrons at the Fe and Ir sites was determined to be ~ 2.4 for IrFe-SACs (*μ*_eff_ = 3.25 *μ*_B_), which suggests that upon coupling of Fe-N_4_ with Ir-N_4_, the Fe centers in the Fe-N_4_ moiety undergo a transition from a low-spin (LS) to a medium-spin (MS) state, involving a mixed valence of Fe^2+^ and Fe^3+^ [39].

### ORR Performance

**Fig. 4:**
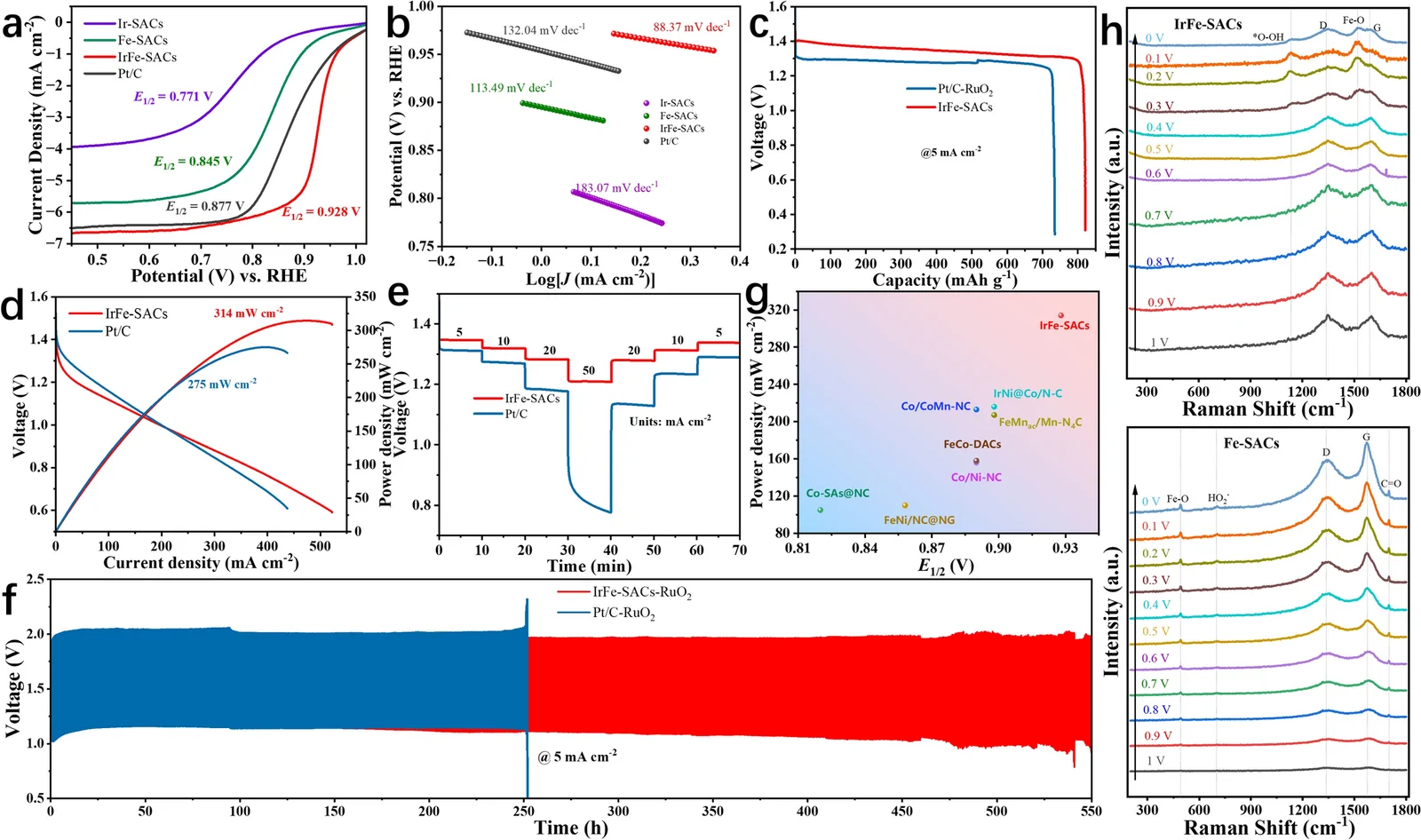
**a** ORR LSV and **b** Tafel curves of IrFe-SACs, Ir-SACs, Fe-SACs and Pt/C. **c** Discharge polarization curves, **d** power density curves, **e** discharge curves at different current densities and** f** galvanostatic charge–discharge cycling curves at 5 mA cm^−2^ of liquid ZABs based on IrFe-SACs-RuO_2_ and Pt/C-RuO_2_ air cathode. **g** Comparison of catalytic and discharge performance between IrFe-SACs and other recently reported oxygen electrocatalysts.** h** In-situ electrochemical Raman spectroscopy of Fe-SACs and IrFe-SACs

The ORR electrocatalytic performance was systematically evaluated using a rotating disk electrode (RDE) system in oxygen-saturated 0.1 M KOH solution at 1600 rpm rotation speed [40]. As shown in Figs. 4a, S10, and S11, the linear sweep voltammetry (LSV) curves reveal that IrFe-SACs exhibits exceptional catalytic activity with an onset potential (*E*_onset_) of 0.956 V vs. RHE and a half-wave potential (*E*_1/2_) of 0.928 V vs. RHE, surpassing the benchmark Pt/C catalyst (*E*_onset_ = 0.908 V, *E*_1/2_ = 0.877 V), various other bimetallic SACs references and IrFe-SACs without 3DOM structures. Figure S12 indicates that IrFe-SACs have a lower electrochemical impedance, which promotes rapid ORR processes. In addition, we also investigated the effect of Ir loadings in IrFe–SACs for ORR activity. As shown in Fig. S13, the results indicate that both excessively high and excessively low Ir loadings lead to a decrease in the intrinsic ORR activity of IrFe-SACs. Furthermore, the ORR performance of the IrFe-SACs exceeds the most recently reported transition metal-based catalysts. (Table S9). Kinetic analysis through *Tafel* plots (Figs. 4b and S14) demonstrates that IrFe-SACs possesses the most favorable reaction kinetics among all the tested catalysts, as evidenced by its lowest Tafel slope of 88.37 mV dec^−1^. In order to clarify the inherent catalytic activity advantage of interlayer IrFe configuration over intralayer IrFe configuration, we prepared a control catalyst through a one-step method, simultaneously introducing Ir and Fe. As shown in Figs. S15, S16 and Table S10, the interlayer IrFe-SACs exhibits significantly enhanced ORR activity, higher TOF, larger ECSA and higher intrinsic catalytic activity compared to that of planar configuration IrFe-SACs obtained one-step method. To elucidate the reaction mechanism, rotating speed-dependent LSV measurements were performed (Fig. S17a). The corresponding *Koutecký*-*Levich* (K-L) plots exhibit excellent linearity (Fig. S17b), from which the electron transfer number (*n*) was calculated to be 3.971, confirming a dominant four-electron oxygen reduction pathway [41].

Catalytic stability is a critical parameter for practical applications. We evaluated the durability of IrFe-SACs and Pt/C through ORR polarization measurements before and after potential cycling (0.1 M KOH, O_2_-purged, 0.615–1.015 V vs. RHE, 50 mV s^−1^, 200 rpm, 25 °C) [42]. The accelerated durability tests revealed superior stability of IrFe-SACs, exhibiting only a 10 mV negative shift in *E*_1/2_, significantly outperforming Pt/C (Δ*E*_1/2_ = 40 mV) (Fig. S18). As presented in Figs. S19, S20 and Table S11, IrFe SACs can maintain a current density of 85% and stable elemental compositionunder I-t tests for 10,000 s, and the Ir and Fe dual-atom sites remain uniformly dispersed on the carbon support, demonstrating outstanding structural robustness during the cycling process. XPS analysis provides insights into the structural evolution during ORR operation. The C 1*s* spectra (Fig. S21a) demonstrate substantial increases in C-O and oxidized carbon peaks, suggesting progressive carbon support oxidation [43]. Notably, the N 1*s* spectra (Fig. S21b) reveal the disappearance of graphitic nitrogen signatures with concurrent enhancement of M-N_*x*_ peaks, indicating activation of additional M–N-C sites during prolonged ORR operation. Furthermore, the Fe 2*p* spectra (Fig. S21c) show partial oxidation from Fe^2+^ to Fe^3+^ to form Fe–O species after stability testing [44]. These synergistic transformations collectively contribute to the enhanced ORR kinetics and durability [45].

### Battery Performance

To assess the practical feasibility of IrFe-SACs, we developed liquid Zn-air batteries utilizing IrFe-SACs as the air cathode, zinc foil as the anode, and a 6.0 M KOH solution containing 0.2 M Zn(CH_3_COO)_2_ as the electrolyte. Commercial Pt/C-RuO_2_ control devices have been produced for benchmarking under the same conditions [46]. As shown in Fig. S22, the IrFe-SACs-based ZAB achieves a significantly higher open-circuit potential (1.52 V) than that of Pt/C-RuO_2_ counterpart (1.32 V). As expected, the Zn-air batteries with IrFe-SACs as air cathode (Fig. 4c, d) exhibits a higher discharge specific capacity of 817.5 mAh g^−1^ at 5 mA cm^−2^, and a higher a peak power density of 314 mW cm^−2^, in comparison to that of Pt-C/RuO_2_ (732.5 mAh g^−1^ and 275 mW cm^−2^). Remarkably, the IrFe-SACs-based Zn-air batteries maintain excellent rate capability and demonstrates cycling stability, operating continuously over 550 h at 5 mA cm^−2^ and 120 h at 10 mA cm^−2^ without significant degradation (Figs. 4e, f and S23). Benchmarking against state-of-the-art catalysts (Fig. 4g and Table S12) reveals that the IrFe-SACs establishes outstanding performance in both ORR activity and Zn-air battery operation.

### ORR Catalytic Mechanism

To elucidate the fundamental ORR mechanisms at atomic precision, we conducted in situ electrochemical *Raman* spectroscopy under operando conditions (0.0–1.0 V vs. RHE, O_2_-saturated 0.1 M KOH). As shown in Fig. 4h, characteristic Fe–O vibrational peaks at 493 and 1519 cm⁻^1^ are clearly observed in Fe-SACs and IrFe-SACs, respectively, confirming that the atomic Fe-N_4_ sites serve as the primary active centers for ORR. Notably, the intensity of the C=O stretching vibration exhibits a progressive enhancement with decreasing reaction potential in Fe-SACs, accompanied by the emergence of a HO_2_⁻ signal at 704 cm⁻^1^. These spectral features suggest the occurrence of carbon support corrosion and partial 2-electron ORR pathway in Fe-SACs [47, 48]. In contrast, the IrFe-SACs spectrum shows no detectable C=O or HO_2_⁻ related signals, demonstrating that the introduction of Ir-N_4_ sites significantly enhances both the structural durability of the catalyst and the selectivity for the 4e^−^ ORR pathway at Fe-N_4_ active centers [49–51]. Most notably, the emergence of *O–OH intermediate vibration (1134 cm^−1^) below 0.3 V in IrFe-SACs provides direct spectroscopic evidence for the *OOH formation step in the associative mechanism.

**Fig. 5:**
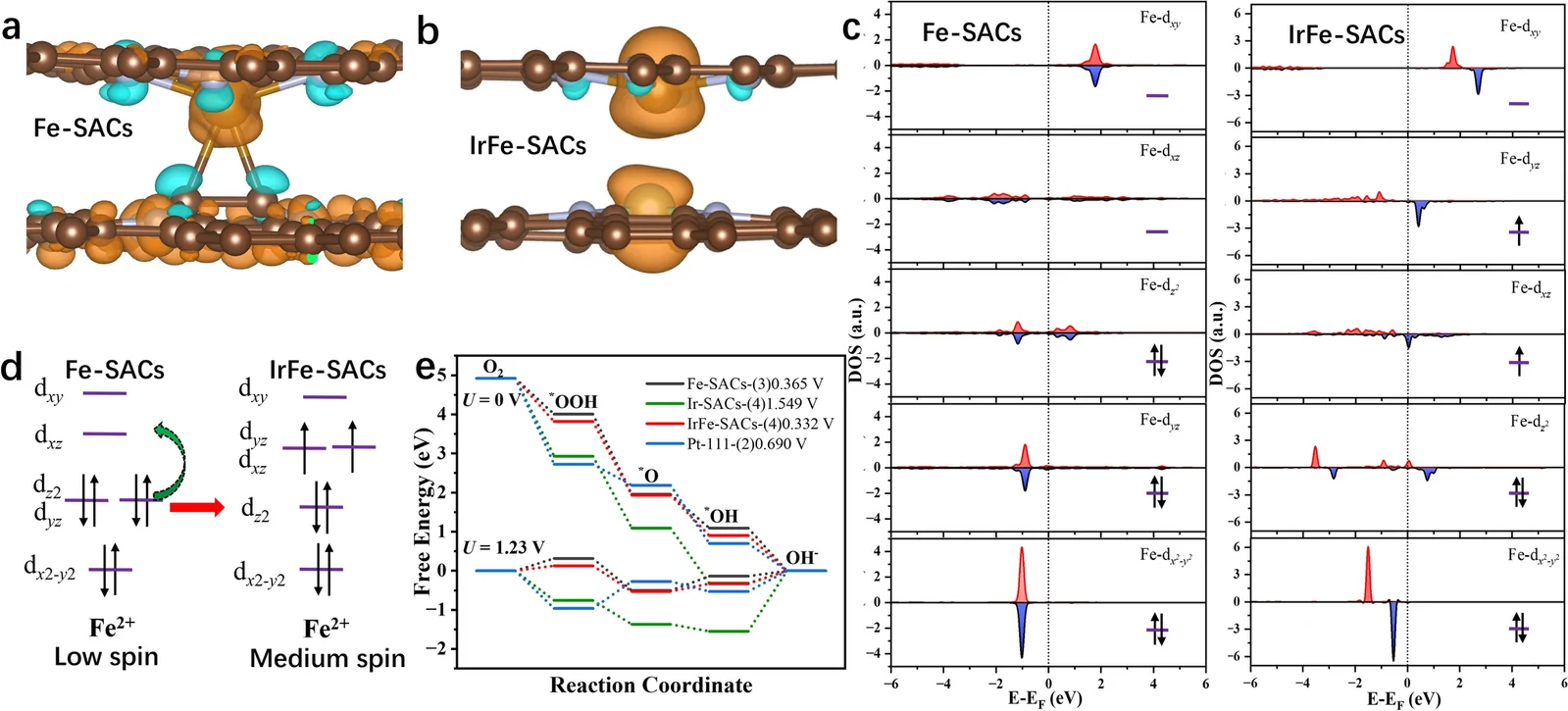
**a, b** Real-space distribution of the spin density (blue and orange colors of the electron clouds represent two different spin orientations), **c** PDOS fine spectra of Fe-d orbitals diagrams, **d** schematic diagrams of the spin-state-energy-level jumps, and **e** ORR path Gibbs free energy step of Fe-SACs and IrFe-SACs

The microenvironment of SACs plays a critical role in modulating orbital splitting, electronic structure, and spin interactions—key factors governing ORR activity [28, 29, 52]. To understand the underlying mechanism in the enhanced ORR activity in IrFe-SACs, we first calculated reaction free energy step diagram, partial density of states (PDOS) and spin states of IrFe-SACs and Fe-SACs (Fig. 5 and Tables S13 and S14). The energy levels of all d-orbital of Fe can be roughly determined from average weighted peak positions of PDOS. The spin density plot in Fig. 5a, b shows that the atomic Fe exhibits a higher spin density distribution (1.337 *μ*_B_) in bimetallic IrFe-SACs than that of Fe SACs alone (~ 0 *μ*_B_), indicating a stronger spin interaction between the atomic Fe sites and oxygen intermediates [53, 54]. The fine spectra of PDOS in Fig. 5c reveal that Fe-SACs and IrFe-SACs exhibit distinct spin-orbital characteristics: degenerated spin orbitals in Fe-SACs versus split spin orbitals in IrFe-SACs. This difference arises from the synergistic interaction between adjacent Ir-N_4_ and Fe-N_4_ atomic sites in the interlayer structure, which induces varying degrees of splitting across all five spin-degenerate Fe-d orbitals in the Fe-N_4_ configuration. Based on the spin state definition, calculation results indicates that the Fe-N_4_ sites in Fe-SACs adopt an almost low-spin state (spin quantum number S = 0), whereas those in IrFe-SACs undergo a transition toward a medium-spin state (S = 1), as schematically illustrated in Fig. 5d. The spin-state transition can be rationalized through crystal field theory: The incorporation of Ir atoms in IrFe-SACs disrupts the spatial symmetry of the Fe-N_4_ sites, thereby enhancing the orbital splitting energy and prompting an energy-level rearrangement of the Fe-d_*xz*_ and Fe-d_*yz*_ orbitals. This electronic restructuring ultimately drives the observed spin-state transition [44, 55]. The elevated spin state of Fe centers in IrFe-SACs facilitates stronger spin interactions, which are instrumental in promoting the rapid adsorption of oxygen intermediates on the catalyst surface and enhancing ORR activity observed in these systems. As expected, the introduction of Ir can greatly enhance the ORR intrinsic activity of atomic Fe (ORR theoretical overpotential: 0.332 V for IrFe-SACs, 1.549 V for Ir-SACs, 0.365 V for Fe-SACs) (Fig. 5e) [51, 56]. In addition, we also calculated the band structure of Ir-SACs and the PDOS of Ir-d. The results in Figs. S24 and S25 indicate that Ir-SACs exhibit little to no magnetism, which is consistent with previous experimental measurements.

**Fig. 6:**
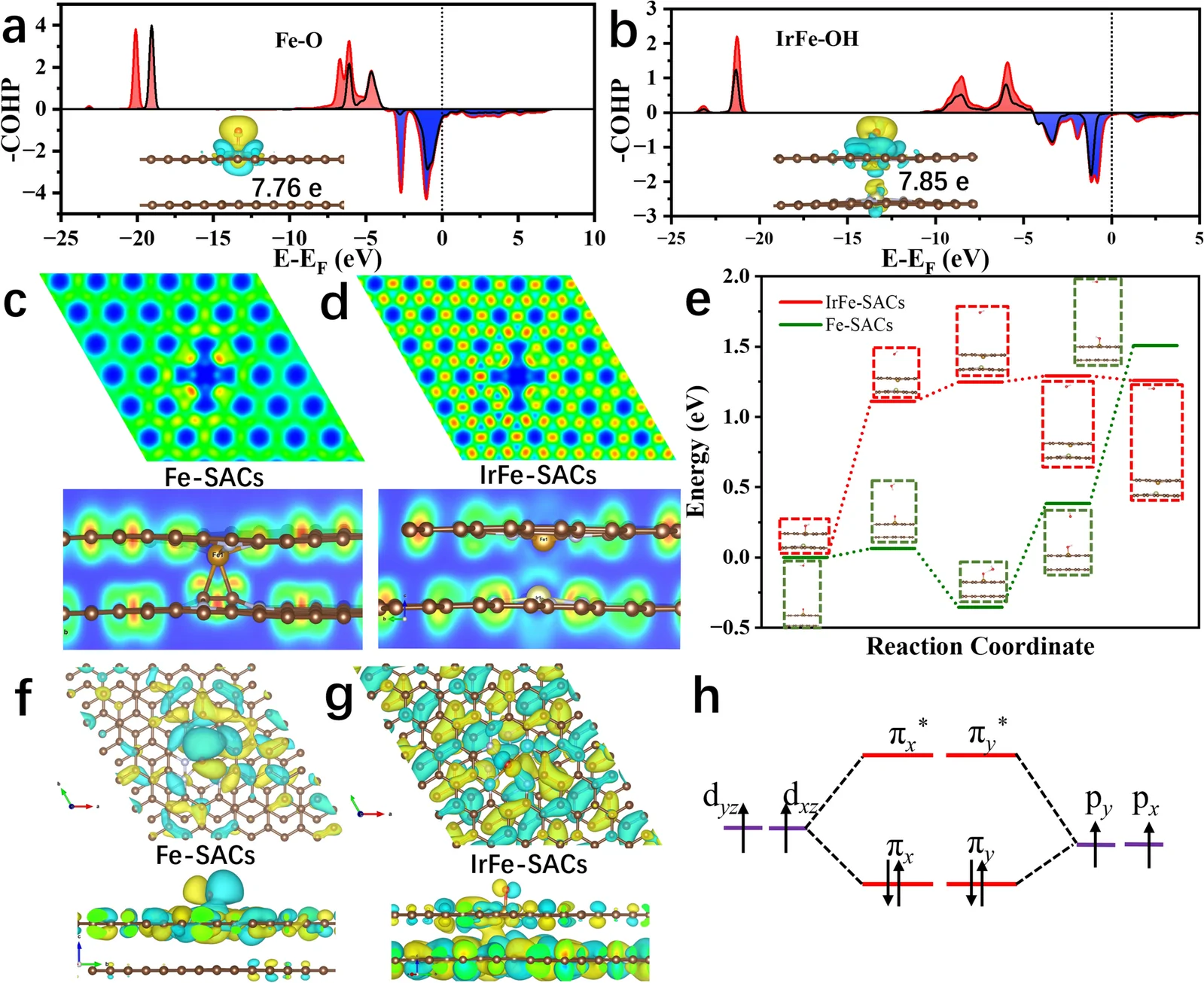
COHP curves (red and black lines represent two different spin orientations) and charge density difference (insets) of Fe-O bonds in **a** Fe-SACs-O and **b** IrFe-SACs-OH. **c**, **d** Electron localization function (ELF) plots for Fe-SACs and IrFe-SACs. **e** Transitionstate step diagrams of oxygen intermediate adsorption by Fe-SACs and IrFe-SACs. **f**, **g** HOMO molecular orbital diagrams of Fe-SACs-O and IrFe-SACs-O. **h** Schematic diagram of Fe-O bond molecular orbitals in Fe-SACs and IrFe-SACs.

To gain deeper insight into the electronic structures under different coordination environments, we conducted a comparative analysis of the charge density difference and oxygen intermediate adsorption behaviors in Fe-SACs and IrFe-SACs. In Fe-SACs, charge redistribution occurs predominantly around the Fe-N_4_ moiety in the outer carbon layer, with the central Fe atom exhibiting a net charge of 7.76 e and an integrated crystal orbital Hamilton population (ICOHP) value of –5.63 for the Fe–O bond. Upon introducing Ir-N_4_ sites, partial charge transfer from the inner-layer Ir-N_4_ to the outer-layer Fe-N_4_ is observed, enhancing the electronegativity of the Fe center. This is evidenced by an increased Fe charge (7.85 e) and a reduced Fe–OH binding strength (ICOHP = − 2.56), indicating weaker adsorption of oxygen intermediates (Fig. 6a, b and Table S4). The increased charge on the Fe active center facilitates efficient electron transfer in the Fe–O bond, thereby enhancing the kinetics of ORR. The adsorption energy results in the Fig. S15 show that IrFe-SACs exhibit more moderate adsorption energy for three oxygen intermediates, indicating its rapid reaction kinetics. Further supporting this trend, the electron localization function (ELF) analysis (Fig. 6c, d) reveals greater charge dispersion near the Fe-N_4_ sites in IrFe-SACs compared to that of Fe-SACs. This electronic redistribution not only strengthens the nonmetallic characteristics of the Fe core but also facilitates rapid nucleophilic reaction and desorption of oxygen intermediates, thus enhancing catalytic activity [36, 57]. To evaluate the kinetic feasibility of the rate-limiting step in ORR, we performed transition state analysis in rate-limiting step to determine the corresponding reaction activation energies (Fig. 6e). These results reveal that the conversion of *O to *OH on Fe-SACs requires overcoming a substantial activation barrier of 1.51 eV, indicating sluggish reaction kinetics. In contrast, the *OH desorption energy barrier on IrFe-SACs is significantly lower (1.25 eV), enabling faster reaction kinetics and improved overall reaction efficiency [58]. To better visualize the adsorption behavior of oxygen intermediates on the atomic catalysts, we further analyzed the electronic band structures and highest occupied molecular orbitals (HOMO) [19, 25]. Further analysis of the molecular orbitals provides additional insights. While both Fe-SACs and IrFe-SACs show Fe-d_*yz*_/O-2p_*y*_ and Fe-d_*xz*_/O-2p_*x*_ orbital hybridization forming π_*y*_ and π_*x*_ orbitals in their *OH/*O adsorbed states the orbital overlap between IrFe-SACs and OH is notably weaker than that in Fe-SACs and O (Figs. 6f-h, S26, and S27). The reduced orbital interaction in IrFe-SACs promotes faster desorption of O/OH intermediates from the catalyst surface, ultimately accelerating the ORR process [17, 19, 59].

According to the thorough experimental and theoretical evaluations we attribute the exceptional ORR activity and stability of the bilayer Ir-N_4_/Fe-N_4_ catalyst to follow factors. First, the incorporation of Ir-N_4_ sites induces spin polarization in Fe-d orbitals, facilitating the transfer of active Fe centers from low-spin state to medium-spin states, leading to enhance spin interactions with oxygen intermediates, and promote their rapid adsorption on catalytic sites. Second, the synergistic interaction between adjacent Ir-N_4_ and Fe-N_4_ moieties facilitates interlayer charge transfer and electron delocalization, as a result in increasing the delocalization character of HOMO electrons in Fe–O bonds, optimizing oxygen intermediate adsorption strength while enabling efficient O/OH adsorption and desorption—a critical factor for accelerating the ORR kinetics. Third, the 3DOM carbon framework ensures high accessibility of atomic active sites, improves mass transport efficiency, and maximizes active site utilization during catalytic processes.

## Conclusions

In summary, through systematic DFT calculations, we have successfully predicted and identified a high-performance bilayer carbon-supported bimetallic atomic electrocatalyst. The rationally designed 3DOM carbon framework anchoring Ir-N_4_/Fe-N_4_ bimetallic single-atom sites was experimentally fabricated, demonstrating exceptional ORR activity and remarkable durability. When employed in Zn-air batteries, the catalyst exhibits outstanding discharge power density and cycling stability. Mechanistic studies reveal that the adjacent interlayer Ir-N_4_ sites induce a spin-state transition in Fe-N_4_ centers from low-spin to medium-spin configuration. This electronic modulation enhances spin polarization of Fe 3d orbitals and increases the non-localization character of Fe–O molecular orbitals, thereby synergistically optimizing both ORR activity and durability of the IrFe-SACs.

## Supplementary Information

Below is the link to the electronic supplementary material.

Supplementary file1 (DOCX 10788 KB)
