# Supplementary material for: Modulation of the Spin State of Atomic Fe-N4 Sites with Interlayer-Adjacent Ir-N4 for Superior ORR Activity
Source: Nanomicro Lett. 2026 Mar 5;18:272. doi: 10.1007/s40820-026-02108-9 (PMC12963600; doi:10.1007/s40820-026-02108-9)
Supplement: Supplementary file 1 — Supplementary file1 (DOCX 10788 KB) [file 40820_2026_2108_MOESM1_ESM.docx]

Supporting Information for

**Modulation of the Spin State of Atomic Fe-N_4_ Sites with Interlayer-Adjacent Ir-N_4_ for Superior ORR Activity**

Yan Tan^1^, Aoshuang Li^1^, Yijie Wang^1^, Xiucai Jiang^1^, Yiwen Cheng^1^, Dongliang Chao^2,^*, Yuzhong Zhang^1,^*, Chuanwei Cheng^1,^*

^1^School of Physics Science and Engineering, Tongji University, Shanghai 200092, P. R. China

^2^Laboratory of Advanced Materials, Aqueous Battery Center, Shanghai Key Laboratory of Molecular Catalysis and Innovative Materials, Electron Microscope Center of Fudan University, Faculty of Chemistry and Materials, Fudan University, Shanghai 200433, P. R. China

* Corresponding authors. Email: [cwcheng@tongji.edu.cn](mailto:cwcheng@tongji.edu.cn) (Chuanwei Cheng); [yzzhang@tongji.edu.cn](mailto:yzzhang@tongji.edu.cn) (Yuzhong Zhang); [chaod@fudan.edu.cn](mailto:chaod@fudan.edu.cn) (Dongliang Chao)

**S1 Electrochemical measurements**

A platinum wire electrode in a hydrogen-filled chamber was used to simulate the reversible hydrogen electrode (RHE) as the working electrode, with a graphite rod as the counter electrode and an Hg/HgO electrode as the reference electrode. The open-circuit potential-time curve was measured over 2000 seconds in 0.1 M KOH solution using this three-electrode system, and the average open-circuit potential obtained was used as the potential correction value for Hg/HgO relative to RHE.

The observed potential and Hg/HgO can be transformed into RHE using the following formula: *E*(RHE) = *E*(Hg/HgO) + 0.880 V.

The *K*-*L* equation was used to determine the number of electron transfers ($n$) that occurred during ORR：

$$\frac{1}{j}=\frac{1}{j_{k}}+\frac{1}{j_{dl}}=\frac{1}{nFkC_{O_{2}}}+\frac{1}{0.62nFC_{O_{2}}D_{O_{2}}^{2/3}\nu^{-1/6}\omega^{1/2}}$$

Where, respectively, $j$, $j_{k}$, and $j_{dl}$ represent the measured current density, kinetic current, and diffusion-limiting current density. $k$ (cm s^-1^) is the heterogeneous rate constant for electron transfer. $F$ stands for the *Faraday* constant (96485 C mol^−1^), and $\nu$ for the kinetic viscosity of electrolyte (1.0 × 10^−2^ cm^2^ s^-1^). The oxygen concentration in 0.1 M KOH (1.2 × 10^−6^ mol cm^−3^) is donated as $C_{O_{2}}$. The diffusion coefficient of oxygen is donated as $D_{O_{2}}$ (1.9 × 10^−5^ cm^2^ s^−1^).

In accordance with previous studies [S1], a carbon paper integrated with a gas diffusion layer supported on nickel foam was employed as the substrate for the gas diffusion electrode half-cell tests. To achieve a homogeneous catalyst loading, 5 mg of the as-prepared catalyst was dispersed into a mixed solution of 10 µL of 5 wt% Nafion solution containing 500 µL ethanol, followed by sonication for 0.5 hours. Subsequently, 100 µL of the catalyst ink was uniformly drop-cast onto the gas diffusion electrode. The catalyst-coated electrode was then transferred to a vacuum oven and dried at 40 °C for 30 minutes. After complete drying, the electrode was assembled into an in-situ electrochemical reaction cell for performance evaluation. During the tests, an Ag/AgCl electrode and a Pt wire were used as the reference electrode and counter electrode, respectively. Pure oxygen was continuously purged into the bottom chamber of the cell. Polarization curves of all catalysts were measured in 1.0 M KOH electrolyte until stable cyclic voltammetry profiles were observed. All potentials were converted to the reversible hydrogen electrode scale.

The TOF of catalysts was obtained by following equations:

$$TOF = \frac{I}{4nF}$$

Where *I* is the reduction current (A) recorded in GDE measurements, *n* is the molar number of atomic Fe sites obtained from ICP, and *F* is the *Faraday* constant.

**S2 Finite element multi-physics field simulation**

A three-dimensional electrochemistry module in COMSOL Multiphysics 6.2 was called to simulate the concentration distribution of the electrolyte at the electrolyte/electrode surface. The 3D nanoparticle was determined to be a regular decahedron with a side length of 1.2 μm. The 3DOM nanoparticle was determined to be a regular decahedron with a side length of 1.2 μm and a pore diameter of 0.2 μm. The conductivity of the IrFe-SACs was set to be 3 × 10^5^ S m^-1^. The ionic conductivity of the electrolyte was determined to be 65 S m^–1^. The diffusion coefficient of oxygen in electrolyte was set to be 1 × 10^-12^ m^2^ s^-1^. An overpotential of 1.0 V was to excite the oxygen transport process.

**S3 DFT calculation details**

The models of IrFe-SACs, CoFe-SACs, NiFe-SACs, PtFe-SACs, RuFe-SACs, MnFe-SACs, MnFe-SACs, CuFe-SACs, Ir-SACs and Fe-SACs were built in the hexagonal cell with lattice constants of 14.76 Å, 14.76 Å, and 35.00 Å. The interaction between neighboring crystal cells can be significantly reduced by the vacuum layer of 35.00 Å. The massive carbon nanosheet substrate was simulated using a 6×6 graphene supercell carbon layer, which was partly substituted by M-N_4_ (M = Co, Fe, Ni, Ru, Cu, Mn, Zn, Pt, Ir) sites.

In our calculations, the cutoff energy of plane wave expansion was employed at 400 eV and 450 eV for structural optimization and derivation of *Gibbs* free energy, respectively. The *Brillouin zone* was sampled using a 4×4×1 *k* mesh by *Gamma*-centered scheme. The electronic ground-state energy converged to lower than 10^-4^ eV for structural optimization and 10^-5^ eV for total energy calculation, and the crystal structure was relaxed to a *Hellmann Feynman* force of less than 0.01 eV Å^−1^.

The computational hydrogen electrode (CHE) model was used to determine the *Gibbs* free energy of each elementary reaction in ORR processes. The four electronic reaction routes listed below can be used to characterize the entire ORR process:

$$\begin{aligned} {*+O}_{2}(g)+H_{2}O(l)+e^{-}\to\mathrm{HOO}^{*}+\mathrm{OH}^{-} \\ \mathrm{HOO}^{*}+e^{-}\to O^{*}+\mathrm{OH}^{-} \\ O^{*}+H_{2}O(l)+e^{-}\to\mathrm{HO}^{*}+\mathrm{OH}^{-} \\ \mathrm{HO}^{*}+e^{-}\to\mathrm{OH}^{-}+* \end{aligned}$$

ORR electrocatalytic properties are commonly described by *Gibbs* free energy change, which is calculated as: $\Delta G=\Delta E+\Delta ZPE-T\Delta S-neU$

where Δ*E* is characterized as the total energy change of system; *n* is electron transfer number in a certain state; ΔZPE and Δ*S* stand for the zero-point energy change and entropy change, respectively, which are based on the calculated vibration frequency of the reactants and products by DS-PAW or the standard table. The *Gibbs* free energy of O_2_ was calculated as $G_{O_{2}}=2G_{H_{2}O}-2G_{H_{2}}-4.92 \mathrm{eV}$ to prevent improper description of the O_2_ triplet state in DFT calculations. The theoretical overpotential (*η*_ORR_ or *η*_OER_) of a reaction was determined as the difference between the voltage needed to convert all free energy steps and the minimal potential (1.23 V) needed for ORR/OER in theory:

$$\eta_{\mathrm{ORR}}=\frac{max(\Delta G_{1-4})+1.23eV}{e}$$

The calculation formula of total density of states center$E_{c}$ is as follows:

$$E_{c}=\frac{\int_{-\infty}^{+\infty} E\rho\left( E \right)dE}{\int_{-\infty}^{+\infty} \rho\left( E \right)dE}$$

where $\rho\left( E \right)$ is the total density of states, and $E$ is corresponding energy eigenvalue.

**Supplementary Figures and Tables**

**
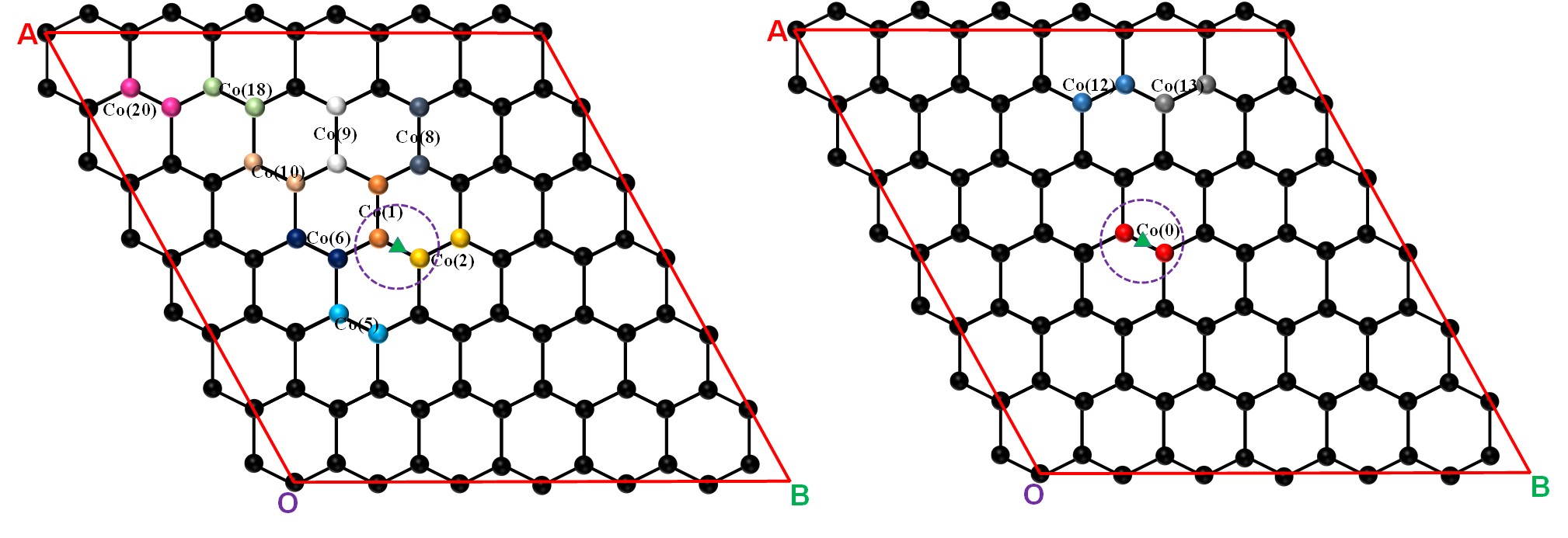
**

**Fig. S1** Partial possible bilayer bimetallic atomic catalyst configurations

**Table S1** Ground state energies (*E*) and diatomic metal spacings (*d*) for various atomic configurations based on DS-PAW

| **Models** | ***d* (Å)** | ***E* (eV)** | **Models** | ***d* (Å)** | ***E*(eV)** |
| --- | --- | --- | --- | --- | --- |
| **Co(1)** | 3.044 | -30201.122 | **Co(11)** | 5.709 | -30201.327 |
| **Co(2)** | 2.727 | -30201.663 | **Co(12)** | 5.860 | -30201.335 |
| **Co(3)** | 4.018 | -30201.292 | **Co(13)** | 5.808 | -30201.321 |
| **Co(4)** | 3.212 | -30201.060 | **Co(14)** | 6.229 | -30201.261 |
| **Co(5)** | 3.193 | -30201.337 | **Co(15)** | 6.275 | -30201.322 |
| **Co(6)** | 3.812 | -30201.301 | **Co(16)** | 6.497 | -30201.048 |
| **Co(7)** | 4.592 | -30200.612 | **Co(17)** | 6.806 | -30201.344 |
| **Co(8)** | 4.749 | -30201.032 | **Co(18)** | 7.580 | -30201.331 |
| **Co(9)** | 5.213 | -30201.056 | **Co(19)** | 8.400 | -30201.328 |
| **Co(10)** | 5.505 | -30201.323 | **Co(20)** | 9.337 | -30201.312 |
| **Co(0)** | 2.511 | -30201.963 |  |  |  |

**Table S2** The calculated results of various oxygen intermediates for different atomic configurations. "*G*" refers to the total *Gibbs* free energy of the whole system (oxygen intermediate + small molecular species) in certain reaction step

| **Items** | | **Models** | | ***(+H_2_+O_2_)** | | ***OOH** | | ***O** | | ***OH** | | ***(+H_2_O)** |  |
| --- | --- | --- | --- | --- | --- | --- | --- | --- | --- | --- | --- | --- | --- |
| ***E*/eV** | | **CoFe-SACs** | | -1309.0965 | | -1323.8386 | | -1314.7630 | | -1319.3238 | | -1309.0965 |  |
| ***ZPE*/eV** | |  |  | 0.0000 | | 0.4286 | | 0.0697 | | 0.3523 | | 0.0000 |  |
| ***TS*/eV** | |  |  | 0.0000 | | 0.1892 | | 0.0624 | | 0.0903 | | 0.0000 |  |
| ***G*/eV** | |  |  | -1332.6165 | | -1333.7993 | | -1335.7757 | | -1336.6818 | | -1337.5365 |  |
| **Δ*G*/eV** | |  |  | 4.9200 | | 3.7372 | | 1.7608 | | 0.8547 | | 0.0000 |  |
| ***U*=1.23 V** | |  |  | 0.0000 | | 0.0472 | | -0.6992 | | -0.3753 | | 0.0000 |  |
| ***E*/eV** | | **NiFe-SACs** | | -1307.4708 | | -1322.5558 | | -1313.5079 | | -1318.0237 | | -1307.4708 |  |
| ***ZPE*/eV** | |  |  | 0.0000 | | 0.4294 | | 0.0690 | | 0.3548 | | 0.0000 |  |
| ***TS*/eV** | |  |  | 0.0000 | | 0.1853 | | 0.0657 | | 0.0908 | | 0.0000 |  |
| ***G*/eV** | |  |  | -1330.9908 | | -1332.5117 | | -1334.5246 | | -1335.3797 | | -1335.9108 |  |
| **Δ*G*/eV** | |  |  | 4.9200 | | 3.3991 | | 1.3862 | | 0.5311 | | 0.0000 |  |
| ***U*=1.23 V** | |  |  | 0.0000 | | -0.2909 | | -1.0738 | | -0.6989 | | 0.0000 |  |
| ***E*/eV** | | **MnFe-SACs** | | -1311.0771 | | -1325.2287 | | -1316.1794 | | -1320.7829 | | -1311.0771 |  |
| ***ZPE*/eV** | |  |  | 0.0000 | | 0.4286 | | 0.0675 | | 0.3412 | | 0.0000 |  |
| ***TS*/eV** | |  |  | 0.0000 | | 0.1911 | | 0.0581 | | 0.1016 | | 0.0000 |  |
| ***G*/eV** | |  |  | -1334.5971 | | -1335.1913 | | -1337.1901 | | -1338.1632 | | -1339.5171 |  |
| **Δ*G*/eV** | |  |  | 4.9200 | | 4.3258 | | 2.3270 | | 1.3539 | | 0.0000 |  |
| ***U*=1.23 V** | |  |  | 0.0000 | | 0.6358 | | -0.1330 | | 0.1239 | | 0.0000 |  |
| ***E*/eV** | | **CuFe-SACs** | | -1304.4016 | | -1319.5540 | | -1310.5284 | | -1315.0305 | | -1304.4016 |  |
| ***ZPE*/eV** | |  |  | 0.0000 | | 0.4280 | | 0.0685 | | 0.3537 | | 0.0000 |  |
| ***TS*/eV** | |  |  | 0.0000 | | 0.1866 | | 0.0664 | | 0.0917 | | 0.0000 |  |
| ***G*/eV** | |  |  | -1327.9216 | | -1329.5126 | | -1331.5464 | | -1332.3886 | | -1332.8416 |  |
| **Δ*G*/eV** | |  |  | 4.9200 | | 3.3290 | | 1.2952 | | 0.4530 | | 0.0000 |  |
| ***U*=1.23 V** | |  |  | 0.0000 | | -0.3610 | | -1.1648 | | -0.7770 | | 0.0000 |  |
| ***E*/eV** | | **RuFe-SACs** | | -1310.1918 | | -1324.5461 | | -1314.9766 | | -1320.0661 | | -1310.1918 |  |
| ***ZPE*/eV** | |  |  | 0.0000 | | 0.4382 | | 0.0679 | | 0.3391 | | 0.0000 |  |
| ***TS*/eV** | |  |  | 0.0000 | | 0.1756 | | 0.0584 | | 0.1125 | | 0.0000 |  |
| ***G*/eV** | |  |  | -1333.7118 | | -1334.4835 | | -1335.9871 | | -1337.4595 | | -1338.6318 |  |
| **Δ*G*/eV** | |  |  | 4.9200 | | 4.1483 | | 2.6447 | | 1.1723 | | 0.0000 |  |
| ***U*=1.23 V** | |  |  | 0.0000 | | 0.4583 | | 0.1847 | | -0.0577 | | 0.0000 |  |
| ***E*/eV** | | **PtFe-SACs** | | -1307.8772 | | -1322.9869 | | -1313.9419 | | -1318.4528 | | -1307.8772 |  |
| ***ZPE*/eV** | |  |  | 0.0000 | | 0.4282 | | 0.0689 | | 0.3550 | | 0.0000 |  |
| ***TS*/eV** | |  |  | 0.0000 | | 0.1891 | | 0.0661 | | 0.0892 | | 0.0000 |  |
| ***G*/eV** | |  |  | -1331.3972 | | -1332.9478 | | -1334.9591 | | -1335.8071 | | -1336.3172 |  |
| **Δ*G*/eV** | |  |  | 4.9200 | | 3.3694 | | 1.3581 | | 0.5101 | | 0.0000 |  |
| ***U*=1.23 V** | |  |  | 0.0000 | | -0.3206 | | -1.1019 | | -0.7199 | | 0.0000 |  |
| ***E*/eV** | | **IrFe-SACs** | | -1310.2555 | | -1324.9336 | | -1315.7519 | | -1320.4409 | | -1310.2555 |  |
| ***ZPE*/eV** | |  |  | 0.0000 | | 0.4348 | | 0.0707 | | 0.3528 | | 0.0000 |  |
| ***TS*/eV** | |  |  | 0.0000 | | 0.1795 | | 0.0598 | | 0.0899 | | 0.0000 |  |
| ***G*/eV** | |  |  | -1333.7755 | | -1334.8784 | | -1336.7611 | | -1337.7979 | | -1338.6955 |  |
| **Δ*G*/eV** | |  |  | 4.9200 | | 3.8171 | | 1.9344 | | 0.8976 | | 0.0000 |  |
| ***U*=1.23 V** | |  |  | 0.0000 | | 0.1271 | | -0.5256 | | -0.3324 | | 0.0000 |  |
| ***E*/eV** | | **ZnFe-SACs** | | -1302.6257 | | -1317.6007 | | -1308.5112 | | -1313.0738 | | -1302.6257 |  |
| ***ZPE*/eV** | |  |  | 0.0000 | | 0.4285 | | 0.0674 | | 0.3524 | | 0.0000 |  |
| ***TS*/eV** | |  |  | 0.0000 | | 0.1878 | | 0.0688 | | 0.0939 | | 0.0000 |  |
| ***G*/eV** | |  |  | -1326.1457 | | -1327.5599 | | -1329.5326 | | -1330.4353 | | -1331.0657 |  |
| **Δ*G*/eV** | |  |  | 4.9200 | | 3.5058 | | 1.5331 | | 0.6304 | | 0.0000 |  |
| ***U*=1.23 V** | |  |  | 0.0000 | | -0.1842 | | -0.9269 | | -0.5996 | | 0.0000 |  |
| **Items** | **Models** | | ***** | | ***OOH** | | ***O** | | ***OH** | | ***** | | |
| ***E*/eV** | **Pt-111** | | -324.6554 | | -340.5743 | | -329.9436 | | -335.1102 | | -324.6554 | | |
| ***ZPE*/eV** |  |  | 0.0000 | | 0.4050 | | 0.0562 | | 0.3332 | | 0.0000 | | |
| ***TS*/eV** |  |  | 0.0000 | | 0.1575 | | 0.0628 | | 0.1105 | | 0.0000 | | |
| ***G*/eV** |  |  | -348.1754 | | -350.3693 | | -350.9074 | | -352.3970 | | -353.0954 | | |
| **Δ*G*/eV** |  |  | 4.9200 | | 2.7261 | | 2.1880 | | 0.6984 | | 0.0000 | | |
| ***U*=1.23 V** |  |  | 0.0000 | | -0.9639 | | -0.2720 | | -0.5316 | | 0.0000 | | |
| ***E*/eV** | **Ir-SACs** | | -1,317.0225 | | -1,332.6671 | | -1,323.4089 | | -1,328.5056 | | -1,317.0225 | | |
| ***ZPE*/eV** |  |  | 0.0000 | | 0.4577 | | 0.0892 | | 0.3840 | | 0.0000 | | |
| ***TS*/eV** |  |  | 0.0000 | | 0.1216 | | 0.0311 | | 0.0396 | | 0.0000 | | |
| ***G*/eV** |  |  | -1,340.5425 | | -1,342.5310 | | -1,344.3708 | | -1,345.7812 | | -1,345.4625 | | |
| **Δ*G*/eV** |  |  | 4.9200 | | 2.9315 | | 1.0917 | | -0.3187 | | 0.0000 | | |
| ***U*=1.23 V** |  |  | 0.0000 | | -0.7585 | | -1.3683 | | -1.5487 | | 0.0000 | | |
| ***E*/eV** | **Fe-SACs** | | -1,317.8284 | | -1,332.3122 | | -1,323.2953 | | -1,327.7780 | | -1,317.8284 | | |
| ***ZPE*/eV** |  |  | 0.0000 | | 0.4163 | | 0.0679 | | 0.3408 | | 0.0000 | | |
| ***TS*/eV** |  |  | 0.0000 | | 0.1658 | | 0.0659 | | 0.1211 | | 0.0000 | | |
| ***G*/eV** |  |  | -1,341.3484 | | -1,342.2617 | | -1,344.31 | | -1,345.1783 | | -1,346.2684 | | |
| **Δ*G*/eV** |  |  | 4.9200 | | 4.0067 | | 1.9552 | | 1.0901 | | 0.0000 | | |
| ***U*=1.23 V** |  |  | 0.0000 | | 0.3167 | | -0.5048 | | -0.1399 | | 0.0000 | | |

**Table S3** The calculated results of various oxygen intermediates for different atomic configurations. "*G*" refers to the total *Gibbs* free energy of the whole system (oxygen intermediate + small molecular species) in certain reaction step

| **Items** | **Models** | ***(+H_2_+O_2_)** | **^*^OOH** | **^*^O** | **^*^OH** | ***(+H_2_O)** |
| --- | --- | --- | --- | --- | --- | --- |
| *E*/eV | FeN_4_C_12_ | -776.6109 | -791.0873 | -782.4278 | -786.8327 | -776.6109 |
| *ZPE*/eV |  | 0.0000 | 0.4194 | 0.0710 | 0.3414 | 0.0000 |
| *TS*/eV |  | 0.0000 | 0.1650 | 0.0660 | 0.1098 | 0.0000 |
| *G*/eV |  | -800.1309 | -801.0329 | -803.4427 | -804.2211 | -805.0509 |
| Δ*G*/eV |  | 4.9200 | 4.0180 | 1.6081 | 0.8298 | 0.0000 |
| *U*=1.23 V |  | 0.0000 | 0.3280 | -0.8519 | -0.4002 | 0.0000 |
| *E*/eV | IrFeN_6_ | -634.3242 | -651.8147 | -641.4821 | -645.2968 | -634.3242 |
| *ZPE*/eV |  | 0.0000 | 0.4488 | 0.0862 | 0.3801 | 0.0000 |
| *TS*/eV |  | 0.0000 | 0.1397 | 0.0371 | 0.0638 | 0.0000 |
| *G*/eV |  | -657.8442 | -661.7056 | -662.4530 | -662.6005 | -662.7642 |
| Δ*G*/eV |  | 4.9200 | 1.0586 | 0.3112 | 0.1637 | 0.0000 |
| *U*=1.23 V |  | 0.0000 | -2.6314 | -2.1488 | -1.0663 | 0.0000 |
| *E*/eV | (Ir)N_4_  FeN_4_ | -632.9697 | -647.3669 | -638.0711 | -642.9731 | -632.9697 |
| *ZPE*/eV |  | 0.0000 | 0.4336 | 0.0596 | 0.3458 | 0.0000 |
| *TS*/eV |  | 0.0000 | 0.1401 | 0.0809 | 0.1293 | 0.0000 |
| *G*/eV |  | -656.4897 | -657.2733 | -659.1124 | -660.3767 | -661.4097 |
| Δ*G*/eV |  | 4.9200 | 4.1363 | 2.2972 | 1.0330 | 0.0000 |
| *U*=1.23 V |  | 0.0000 | 0.4463 | -0.1628 | -0.1970 | 0.0000 |
| *E*/eV | IrN_4_  (Fe)N_4_ | -632.9697 | -647.9507 | -639.0275 | -643.4613 | -632.9697 |
| *ZPE*/eV |  | 0.0000 | 0.4320 | 0.0667 | 0.3258 | 0.0000 |
| *TS*/eV |  | 0.0000 | 0.1776 | 0.0689 | 0.1312 | 0.0000 |
| *G*/eV |  | -656.4897 | -657.8963 | -660.0497 | -660.8867 | -661.4097 |
| Δ*G*/eV |  | 4.9200 | 3.5133 | 1.3600 | 0.5229 | 0.0000 |
| *U*=1.23 V |  | 0.0000 | -0.1767 | -1.1000 | -0.7071 | 0.0000 |
| *E*/eV | (Ir)Fe-SACs | -1293.4728 | -1308.2833 | -1299.1433 | -1303.8073 | -1293.4728 |
| *ZPE*/eV |  | 0.0000 | 0.4328 | 0.0718 | 0.3561 | 0.0000 |
| *TS*/eV |  | 0.0000 | 0.1979 | 0.0584 | 0.0870 | 0.0000 |
| *G*/eV |  | -1316.9928 | -1318.2484 | -1320.1499 | -1321.1582 | -1321.9128 |
| Δ*G*/eV |  | 4.9200 | 3.6644 | 1.7629 | 0.7546 | 0.0000 |
| *U*=1.23 V |  | 0.0000 | -0.0256 | -0.6971 | -0.4754 | 0.0000 |
| *E*/eV | Ir(Fe)-SACs | -1294.3531 | -1308.9473 | -1299.7478 | -1304.5580 | -1294.3531 |
| *ZPE*/eV |  | 0.0000 | 0.4383 | 0.0725 | 0.3581 | 0.0000 |
| *TS*/eV |  | 0.0000 | 0.1813 | 0.0559 | 0.0840 | 0.0000 |
| *G*/eV |  | -1317.8731 | -1318.8904 | -1320.7512 | -1321.9039 | -1322.7931 |
| Δ*G*/eV |  | 4.9200 | 3.9027 | 2.0419 | 0.8892 | 0.0000 |
| *U*=1.23 V |  | 0.0000 | 0.2127 | -0.4181 | -0.3408 | 0.0000 |
| *E*/eV | (IrFe)-SACs | -1,277.5261 | -1,292.1958 | -1,283.0426 | -1,287.7440 | -1,277.5261 |
| *ZPE*/eV |  | 0.0000 | 0.4506 | 0.0718 | 0.3596 | 0.0000 |
| *TS*/eV |  | 0.0000 | 0.1661 | 0.0578 | 0.0840 | 0.0000 |
| *G*/eV |  | -1,301.0461 | -1,302.1113 | -1,304.0485 | -1,305.0883 | -1,305.9661 |
| Δ*G*/eV |  | 4.9200 | 3.8548 | 1.9176 | 0.8778 | 0.0000 |
| *U*=1.23 V |  | 0.0000 | 0.1648 | -0.5424 | -0.3522 | 0.0000 |

**
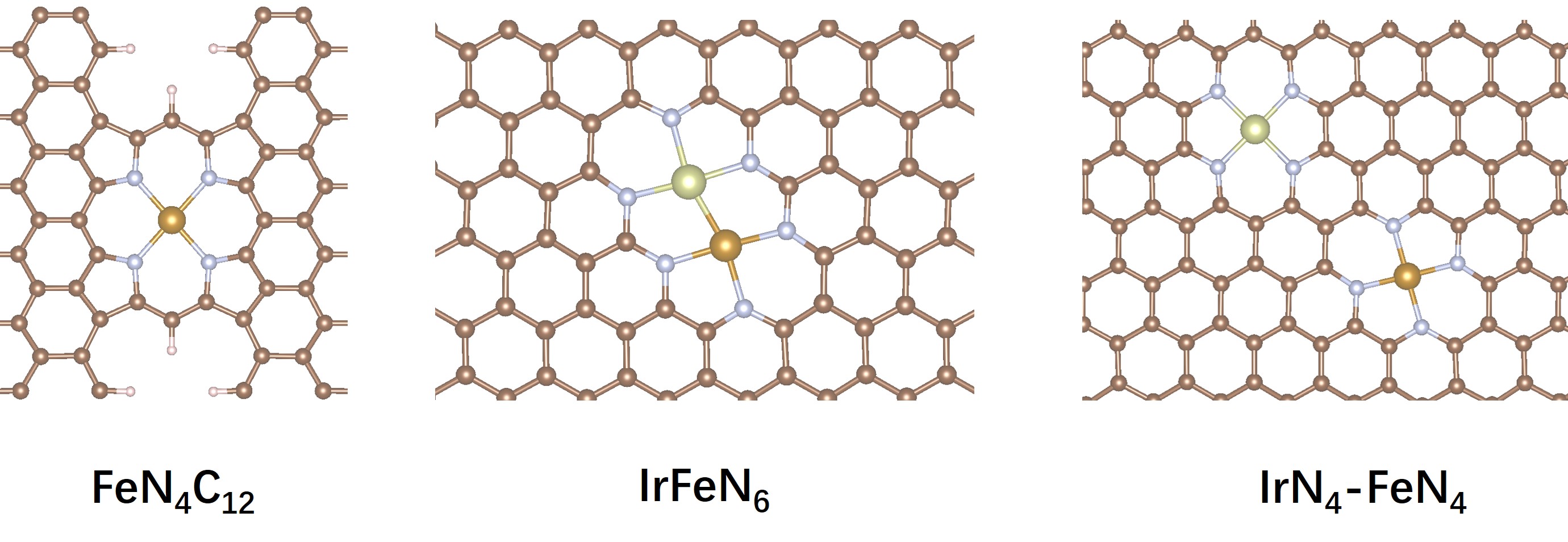
**


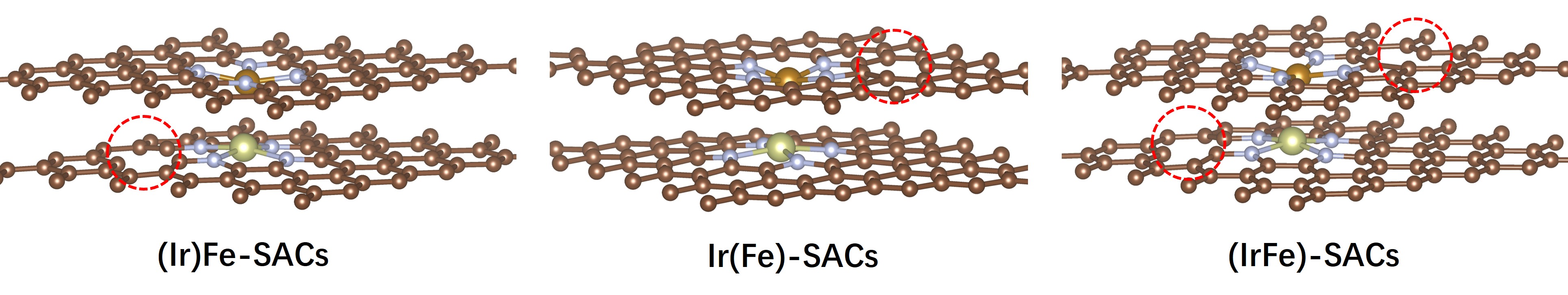


**Fig. S2** Structural diagrams of FeN_4_C_12_, IrFeN_6_, IrN_4_FeN_4_, (Ir)Fe-SACs, Ir(Fe)-SACs and (IrFe)-SACs

**
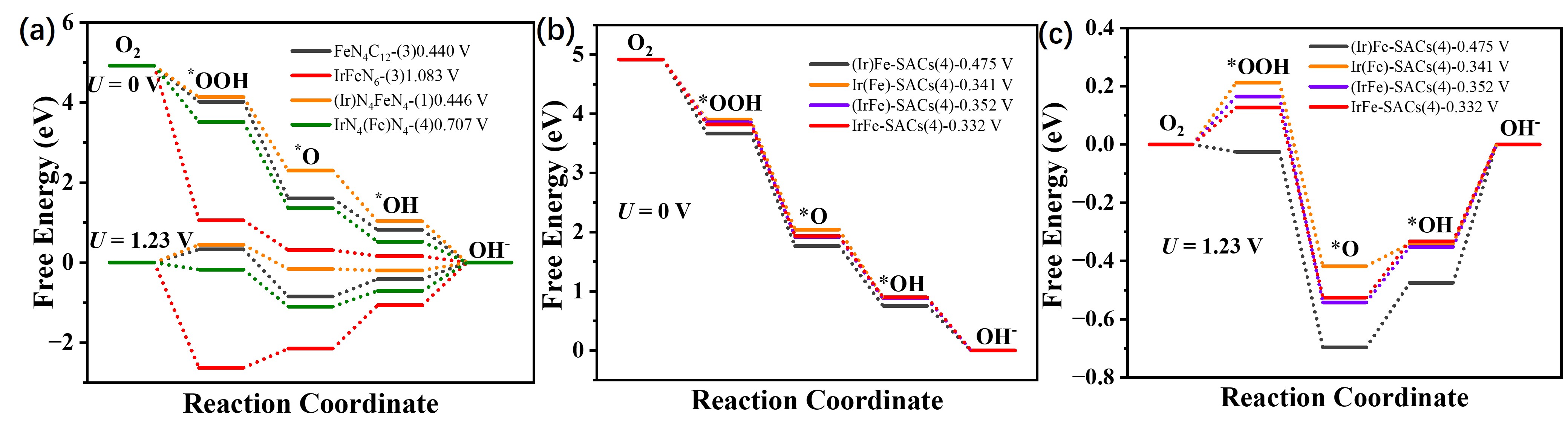
**

**Fig. S3** ORR path *Gibbs* free energy step of FeN_4_C_12_, IrFeN_6_, (Ir)N_4_FeN_4_ (The active center is the atomic Ir), IrN_4_(Fe)N_4_ (The active center is the atomic Fe), (Ir)Fe-SACs, Ir(Fe)-SACs and (IrFe)-SACs

**Table S4** ICOHP for different atomic configurations

| **Spin State** | **Models** | **O*** | **HO*** | **HOO*** |
| --- | --- | --- | --- | --- |
| 1 | IrFe-SACs | -1.9102 | -1.4807 | -1.2780 |
| 2 |  | -3.5731 | -1.0821 | -1.6895 |
| 1 | RuFe-SACs | -1.6668 | -0.9573 | -0.9500 |
| 2 |  | -3.0606 | -1.1996 | -1.1152 |
| 1 | PtFe-SACs | -3.0753 | -4.0312 | -1.5122 |
| 2 |  | -3.0802 | -4.0300 | -2.0255 |
| 1 | NiFe-SACs | -2.1302 | -1.2268 | -1.5092 |
| 2 |  | -3.7579 | -1.7413 | -2.0431 |
| 1 | MnFe-SACs | -1.7247 | -0.9932 | -0.6533 |
| 2 |  | -3.1381 | -0.7523 | -0.9395 |
| 1 | CuFe-SACs | -2.0149 | -1.1944 | -1.4718 |
| 2 |  | -3.7351 | -1.7381 | -2.0315 |
| 1 | CoFe-SACs | -1.9923 | -1.5796 | -1.3350 |
| 2 |  | -3.6618 | -1.1384 | -1.9136 |
| 1 | ZnFe-SACs | -2.0331 | -1.1327 | -2.0303 |
| 2 |  | -3.7104 | -1.6999 | -1.4426 |
| 1 | Fe-SACs | -1.95703 | -1.7825 | -2.12297 |
| 2 |  | -3.67461 | -1.21039 | -1.53651 |
| 1 | Ir-SACs | -3.98811 | -5.40549 | -5.00848 |
| 2 |  | -3.9878 | -5.40551 | -5.00845 |


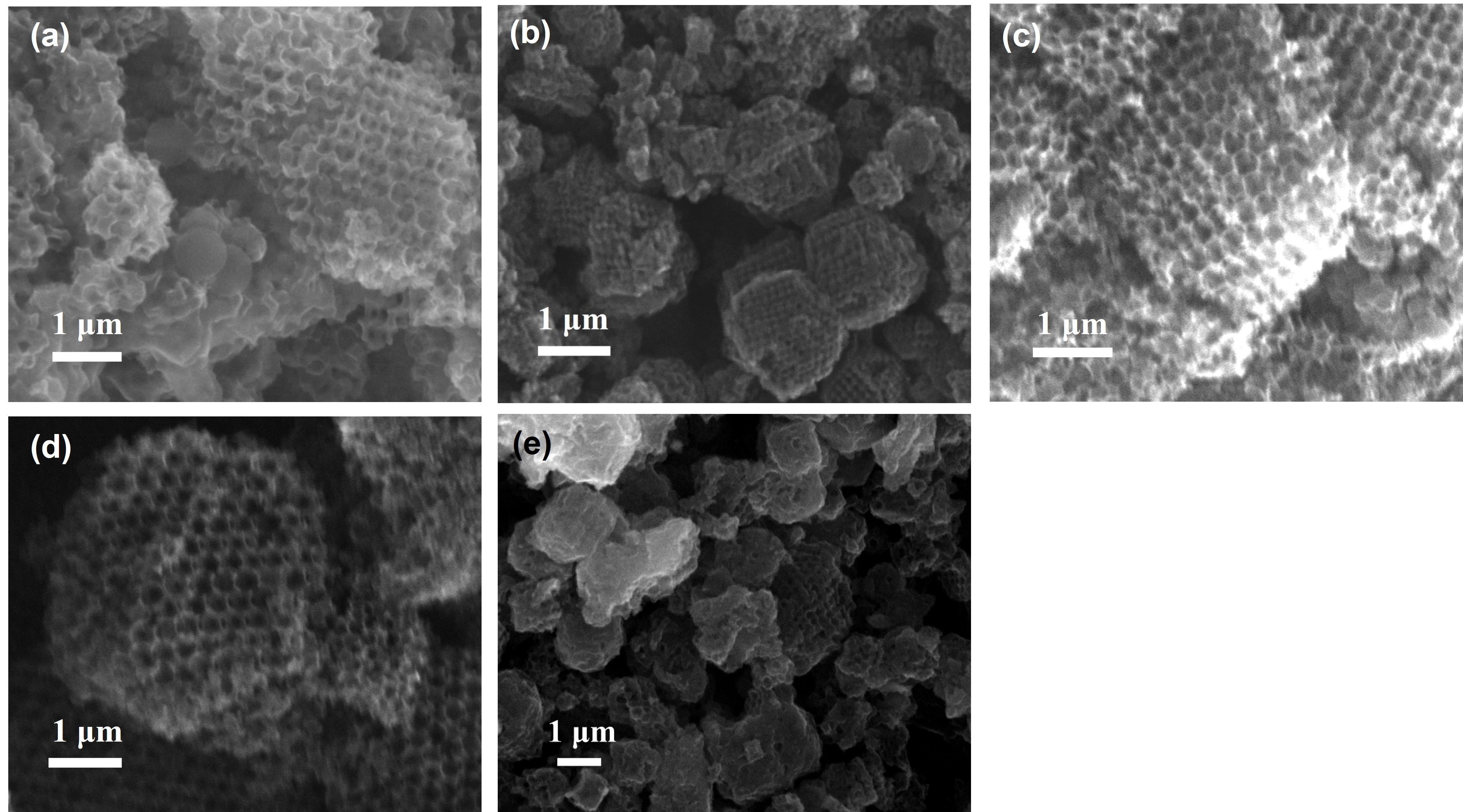


**Fig. S4** SEM images of **(a)** NiFe-SACs, **(b)** CoFe-SACs, **(c)** MnFe-SACs, **(d)** RuFe-SACs and **(e)** CuFe-SACs

**
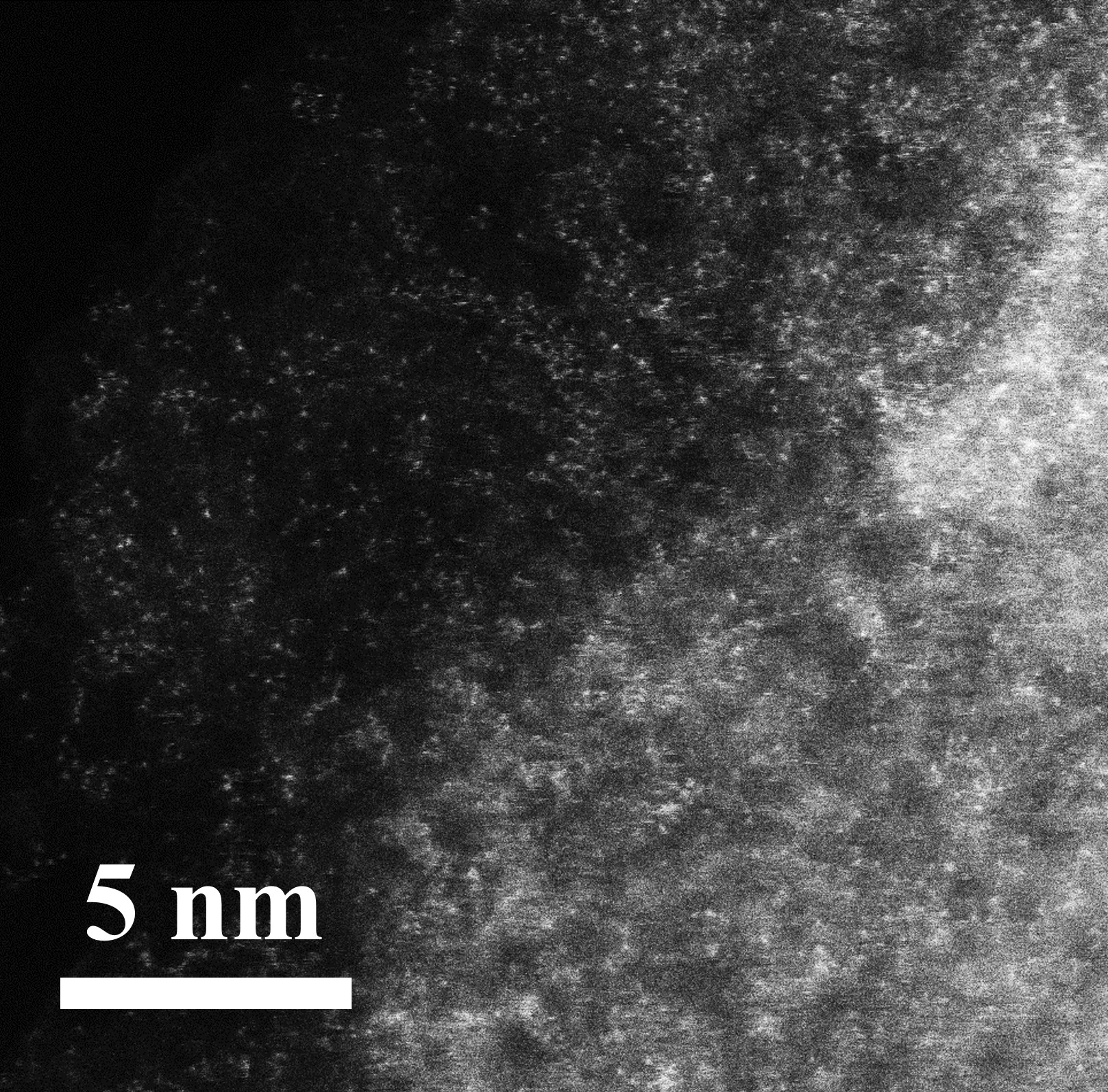
**

**Fig. S5** HAADF-STEM images of IrFe-SACs

**Table S5** EDS table of IrFe-SACs

| **Z** | **Element** | **Family** | **Atomic Fraction (%)** | **Mass Fraction (%)** |
| --- | --- | --- | --- | --- |
| 6 | C | K | 91.84 | 89.20 |
| 7 | N | K | 4.20 | 4.75 |
| 8 | O | K | 3.76 | 4.87 |
| 26 | Fe | K | 0.17 | 1.79 |
| 77 | Ir | M | 0.02 | 0.39 |

**Table S6** ICP results of IrFe-SACs

| **Samples** | **Mass**  **/g** | **Volume**  **/mL** | **Element** | **Readout**  **/mg L^-1^** | **Content**  **/mg kg^-1^** | **wt.%** |
| --- | --- | --- | --- | --- | --- | --- |
| **IrFe-SACs** | 0.0104 | 25 | Fe | 8.5941 | 20658.9 | 2.0659% |
|  | 0.0104 | 25 | Ir | 1.8775 | 4513.2 | 0.4513% |

**Fig. S6** XRD pattern of IrFe-SACs


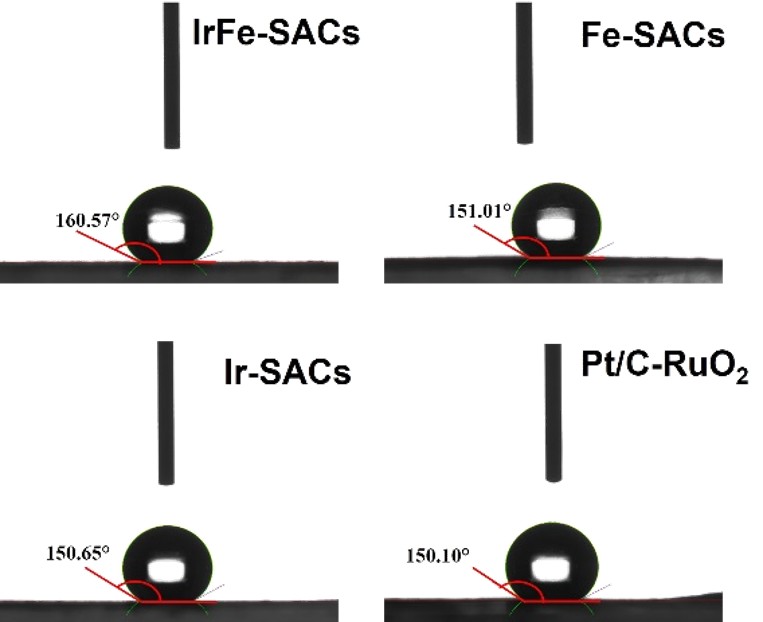


**Fig. S7** Surface contact angles measured at IrFe-SACs, Fe-SACs, Ir-SACs and Pt/C-RuO_2_ electrodes

**
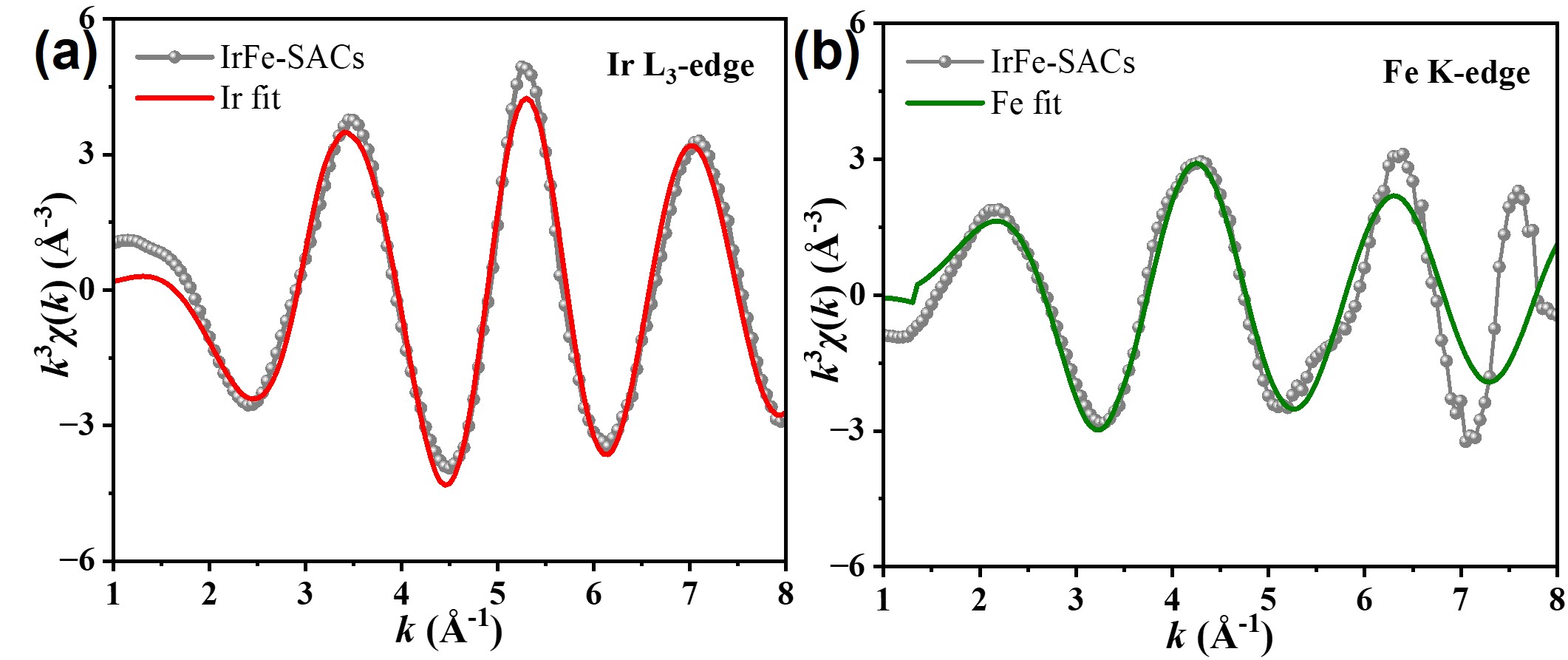
**

**Fig. S8** *Fourier*-transformed *k*^3^-weighted EXAFS fitting results for IrFe-SACs at *k* space

**Table S7** Parameters obtained for the best EXAFS fitting of IrFe-SACs

| **Edges** | **Paths** | ***N*** | ***R*(Å)** | ***σ*^2^ (10^-3^ Å^2^)** | **Happiness** | ***R*-factor** |
| --- | --- | --- | --- | --- | --- | --- |
| Ir L_3_-edge | Ir-N | 3.61±0.05 | 1.95±0.062 | 0.004 | 90.76 | 0.009 |
|  | Ir-Fe | 0.95±0.14 | 2.48±0.013 | 0.034 |  |  |
| Fe K-edge | Fe-N | 3.76±0.01 | 1.89±0.017 | 0.001 | 95.13 | 0.021 |
|  | Fe-Ir | 1.01±0.05 | 2.48±0.028 | 0.013 |  |  |

Where *N* is the coordination number; *R* is the distance between absorber and backscatter atoms; *σ*^2^

is the *Debye*-*Waller* factor (a measure of thermal and static disorder in absorber-scatterer distances);

*R*-factor is used to value the goodness of the fitting.


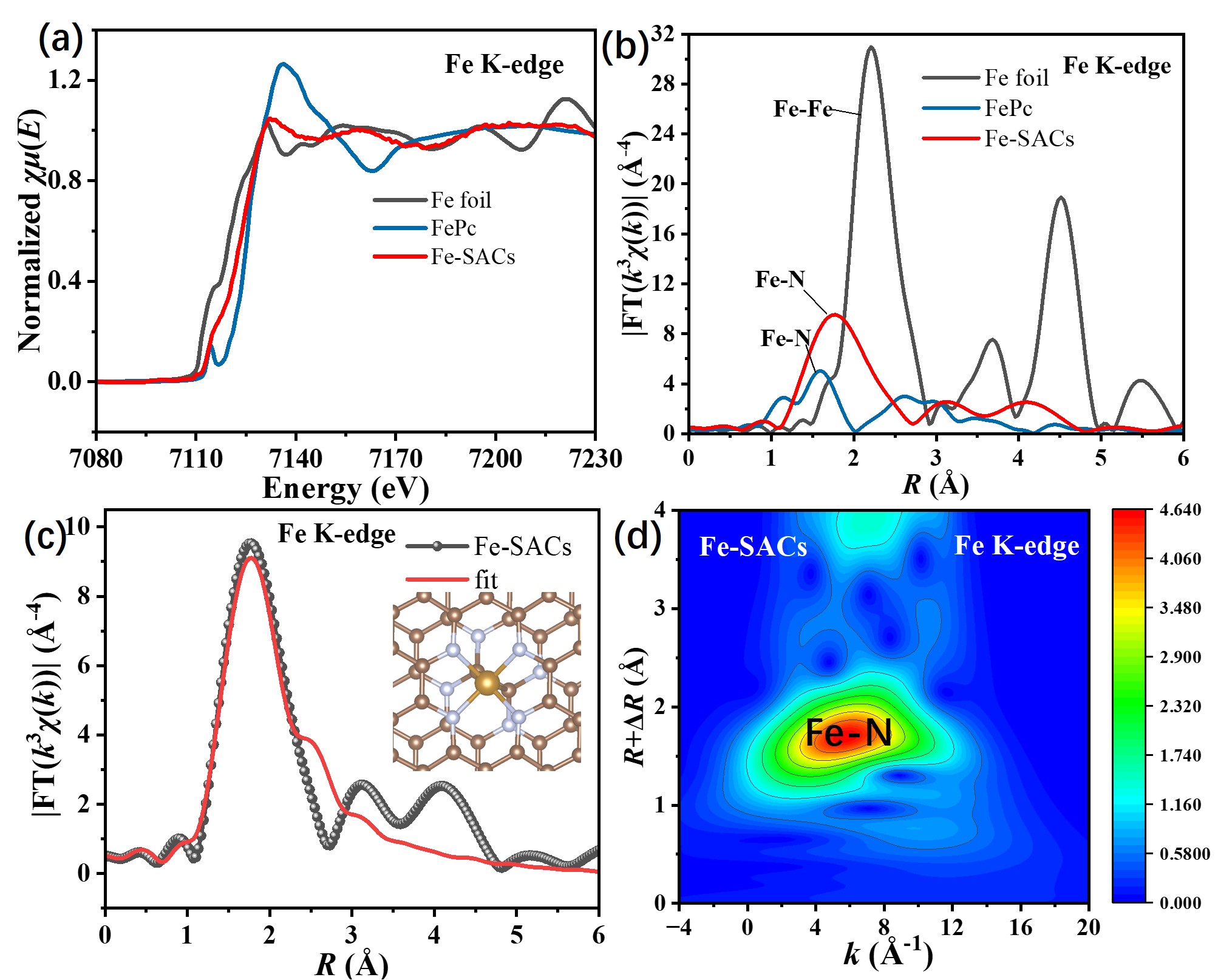


**Fig. S9 (a)** Normalized XANES spectra, **(b)** *Fourier* transforms of EXAFS spectra Fe K-edge of IrFe-SACs and the reference materials at *R* space. XAFS curves represent fitted data based on **(c)** Fe K-edge experimental data, with the fitted curves of Fe-SACs at the insets. **(d)** Wavelet transform of Fe K-edge EXAFS spectra for Fe-SACs

**Table S8** Parameters obtained for the best EXAFS fitting of Fe-SACs

| **Edges** | **Paths** | ***N*** | ***R*(Å)** | ***σ*^2^ (10^-3^ Å^2^)** | **Happiness** | ***R*-factor** |
| --- | --- | --- | --- | --- | --- | --- |
| Fe K-edge | Fe-N | 4.09±0.03 | 2.02±0.021 | 0.006 | 86.52 | 0.011 |
|  | Fe-C | 0.98±0.02 | 2.58±0.018 | 0.015 |  |  |

Where *N* is the coordination number; *R* is the distance between absorber and backscatter atoms; *σ*^2^

is the *Debye*-*Waller* factor (a measure of thermal and static disorder in absorber-scatterer distances);

*R*-factor is used to value the goodness of the fitting.

**
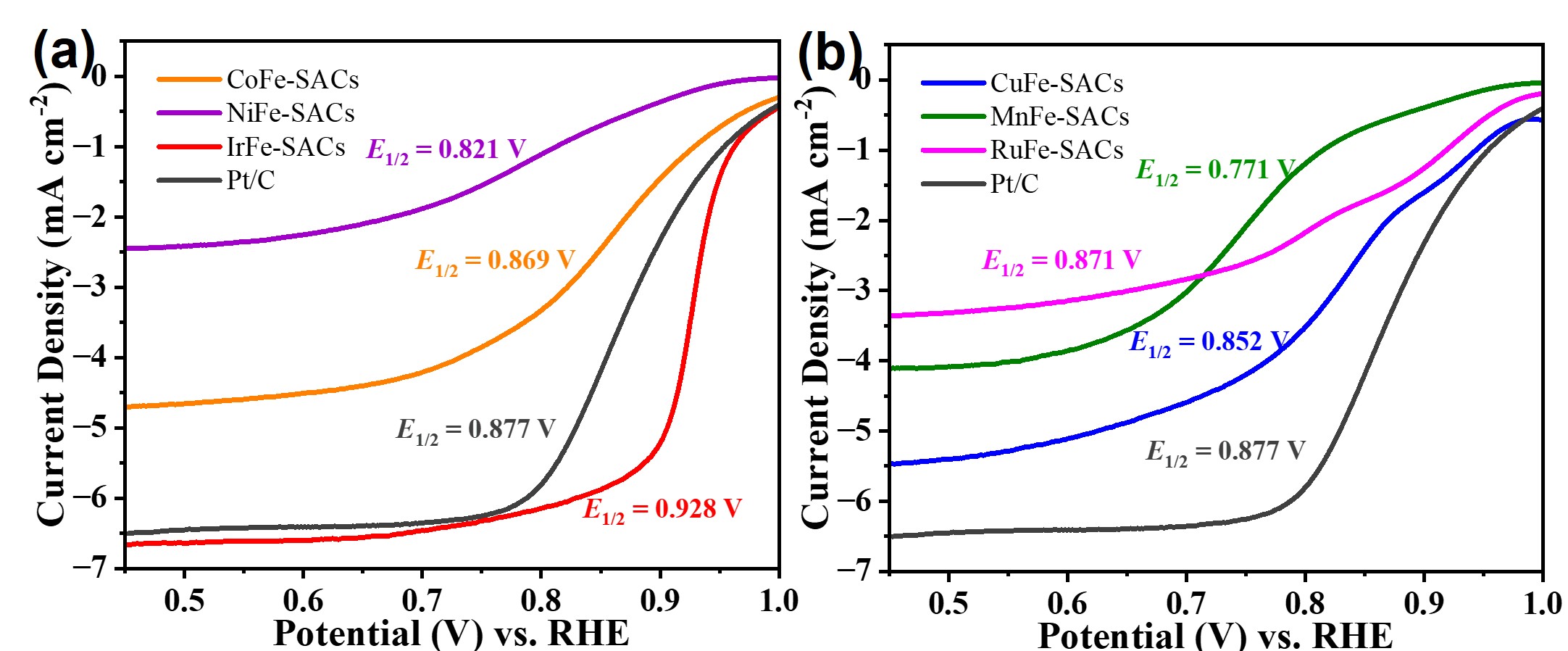
**

**Fig. S10** ORR LSV curves of CuFe-SACs, MnFe-SACs, RuFe-SACs, CoFe-SACs, NiFe-SACs, IrFe-SACs and Pt/C

**
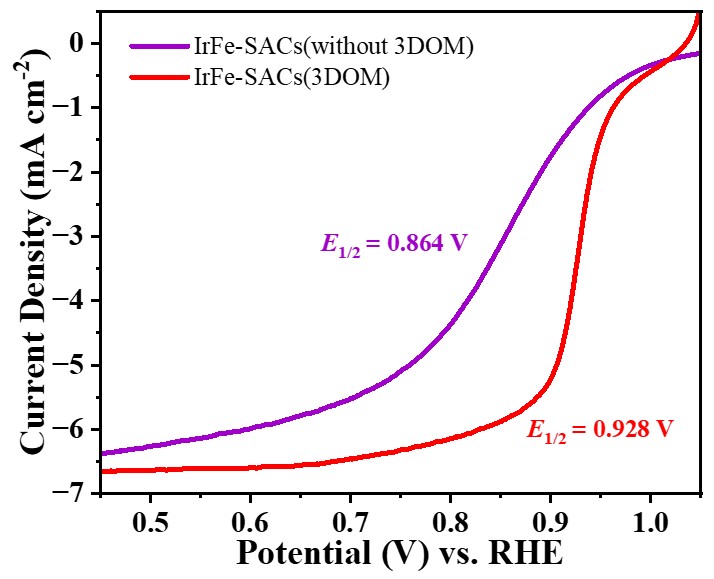
**

**Fig. S11** ORR LSV curves of 3DOM IrFe-SACs and IrFe-SACs without 3DOM structures

**Fig. S12** EIS spectrum of various bimetallic atomic catalysts

**
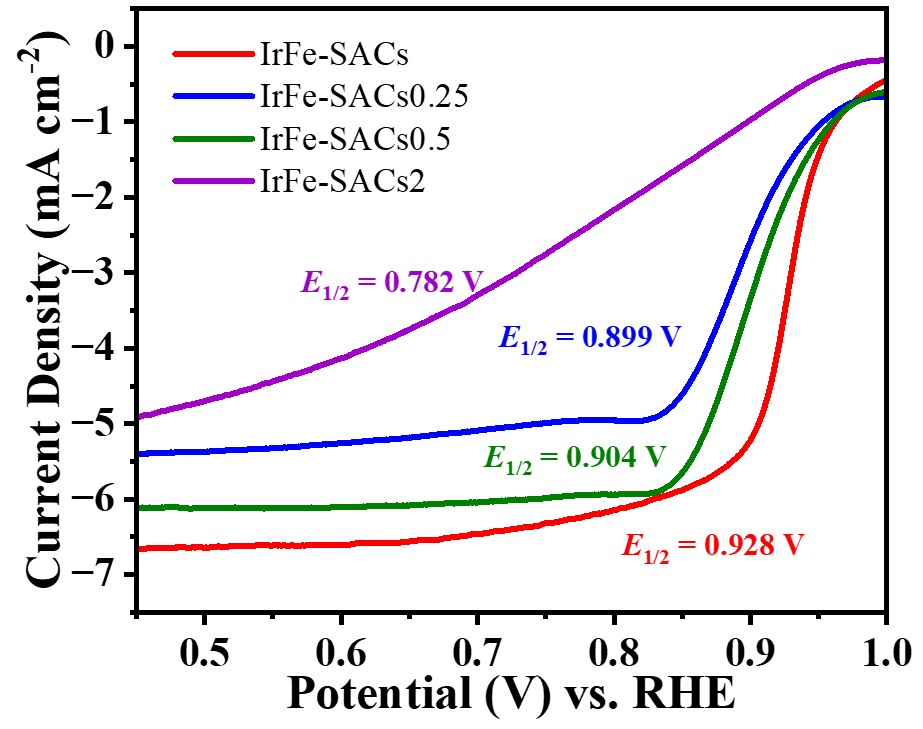
**

**Fig. S13** ORR LSV curves of IrFe-SACs with different Ir loadings. Here, the "x" in "IrFe-SACsx" denotes the feeding ratio of iridium acetylacetonate during the sample preparation, relative to that used for IrFe-SACs

**Table S9** Comparison the ORR activity of IrFe-SACs with the reported other transition metal atomic electrocatalysts

| **Catalysts** | ***E*_onset_ (V vs. RHE)** | | ***E*_1/2_ (V vs. RHE)** | | **References** |
| --- | --- | --- | --- | --- | --- |
| **IrFe-SACs** | **0.961** | **0.928** | | **This work** | |
| Co/CoMn-NC | 0.99 | 0.89 | | [S2] | |
| Co-SAs@NC | 0.96 | 0.82 | | [S3] | |
| Fe_SA_-N-C | 1.00 | 0.892 | | [S4] | |
| Co/Ni-NC | 0.951 | 0.89 | | [S5] | |
| FeNi/NC@NG | 0.949 | 0.858 | | [S6] | |
| IrNi@Co/N-C | 0.973 | 0.898 | | [S7] | |
| FeCo-DACs | 0.915 | 0.89 | | [S8] | |
| Fe_2_DAC | 1.00 | 0.898 | | [S9] | |
| FeCo-NCH | 0.995 | 0.889 | | [S10] | |


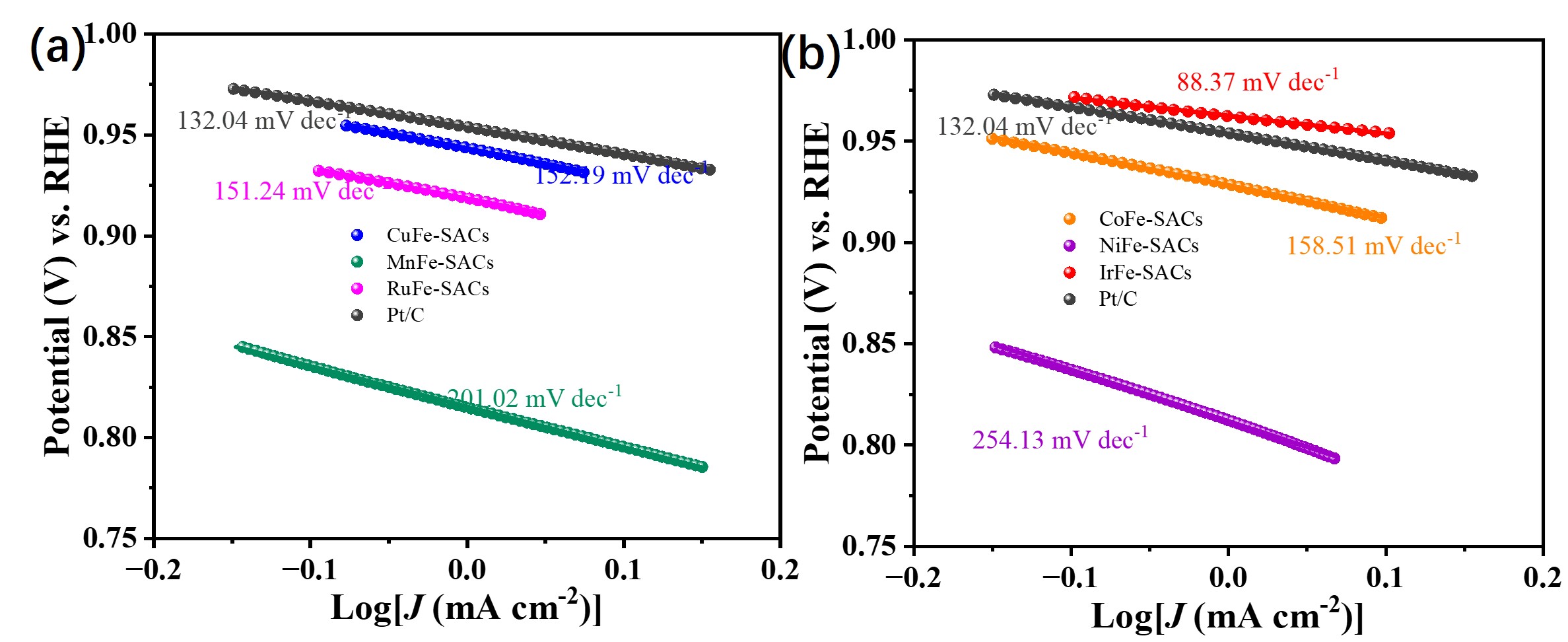


**Fig. S14** *Tafel* curves of CuFe-SACs, MnFe-SACs, RuFe-SACs, CoFe-SACs, NiFe-SACs, IrFe-SACs and Pt/C during ORR tests


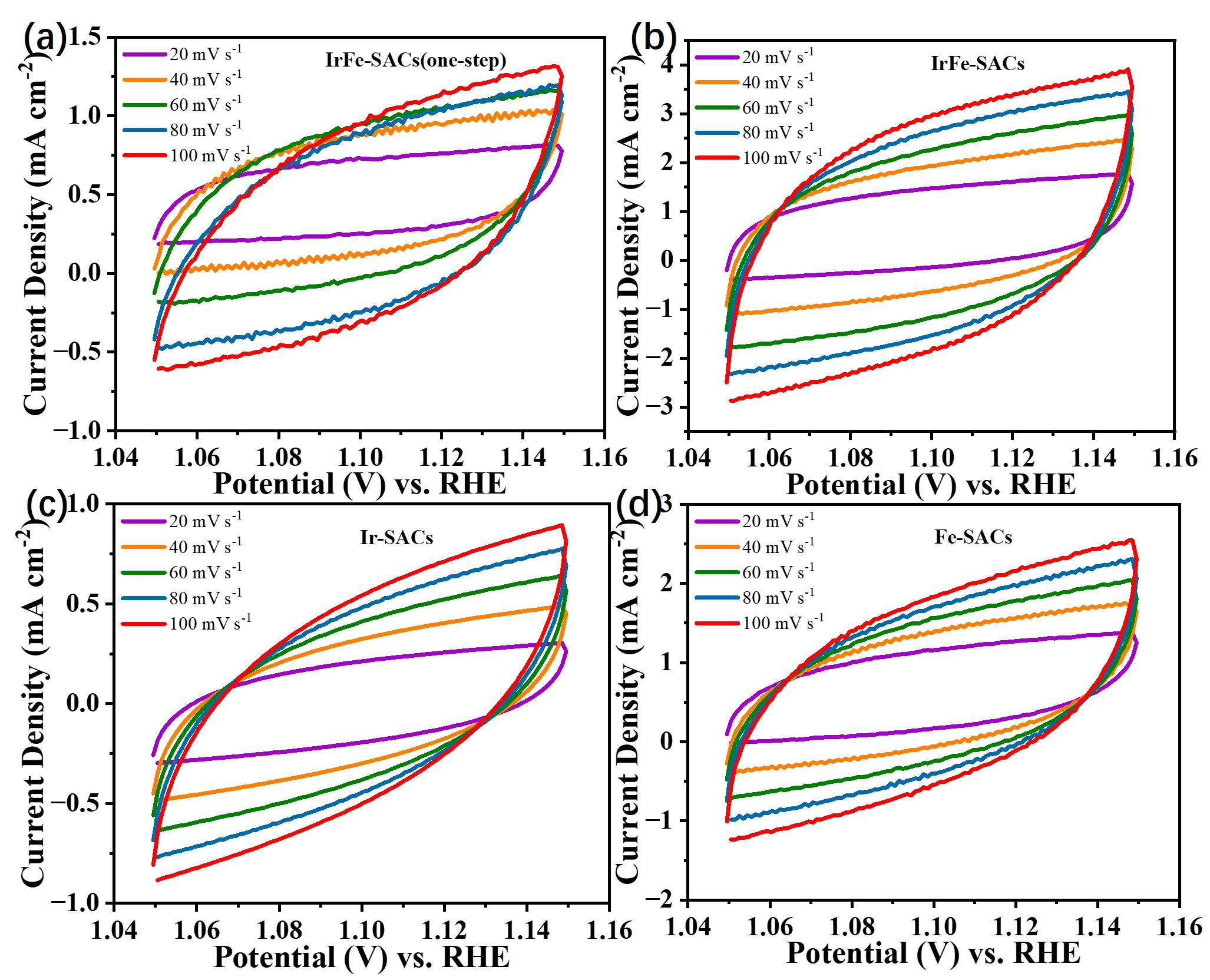


**Fig. S15** Cyclic voltammograms from 1.05 V to 1.15 V vs. RHE in 0.1 M KOH at scan rates of 20, 40, 60, 80 and 100 mV s^-1^ for **(a)** one-step synthesized IrFe-SACs, **(b)** IrFe-SACs, **(c)** Ir-SACs and **(d)** Fe-SACs, respectively

**Table S10** ICP results of IrFe-SACs(one-step)

| **Samples** | **Mass**  **/g** | **Volume**  **/mL** | **Element** | **Readout**  **/mg L^-1^** | **Content**  **/mg kg^-1^** | **wt.%** |
| --- | --- | --- | --- | --- | --- | --- |
| **IrFe-SACs**  **(one-step)** | 0.0218 | 20 | Fe | 35.5268 | 32593.4 | 3.2593% |
|  | 0.0218 | 20 | Ir | 7.9954 | 7335.2 | 0.7335% |


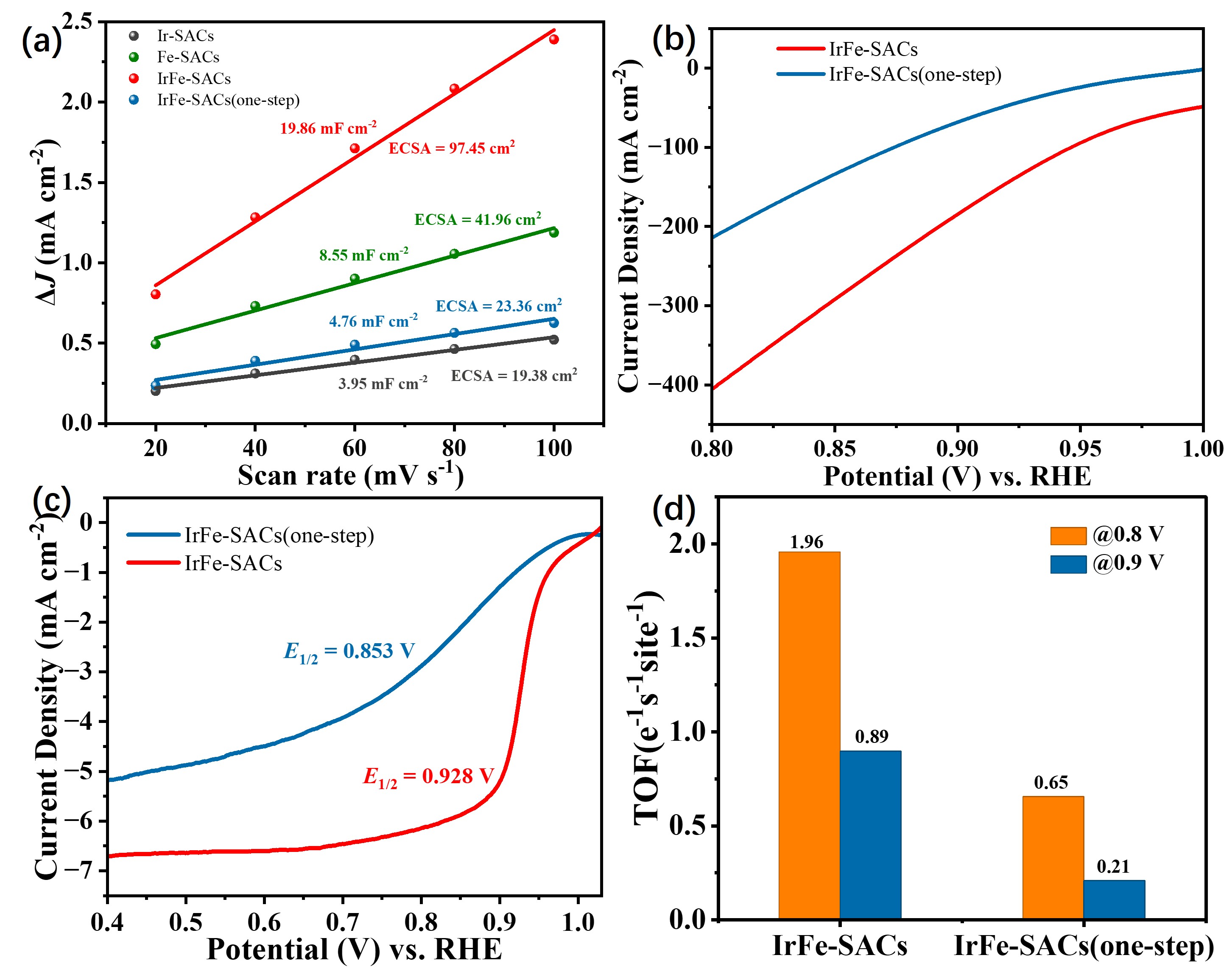


**Fig. S16 (a)** Relations between current density and scanning rate of CV curves between 1.05 and 1.15 V of one-step synthesized IrFe-SACs, IrFe-SACs, Ir-SACs and Fe-SACs. ORR LSV polarization curves in **(b)** 1.0 M KOH with GDEs and **(c)** 0.1 M KOH with RDEs, **(d)** TOF for one-step synthesized IrFe-SACs and IrFe-SACs


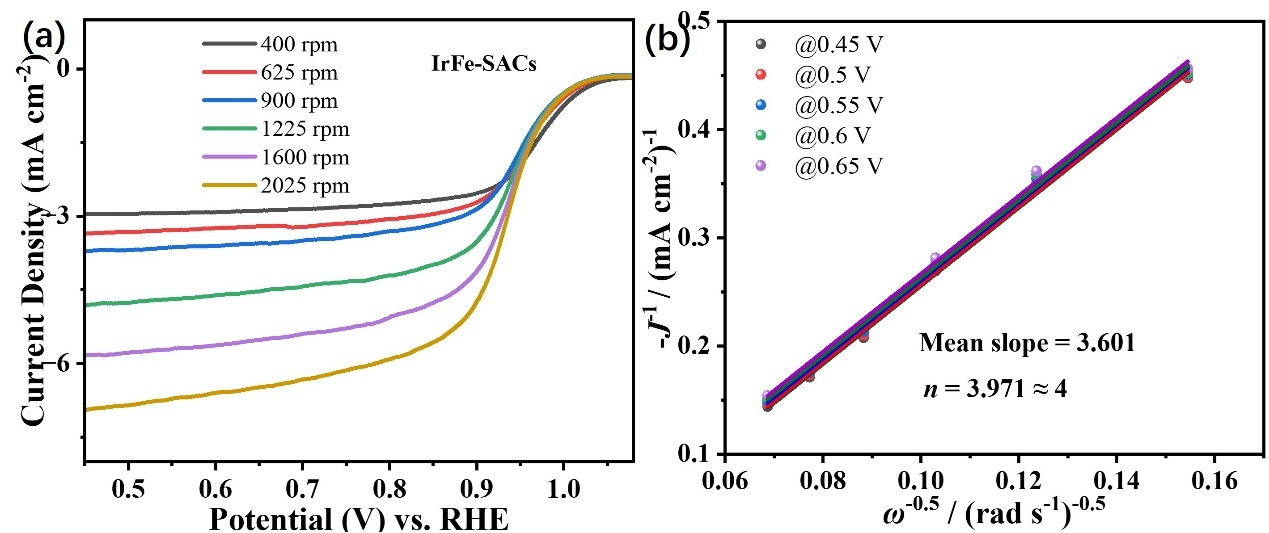


**Fig. S17 (a)** ORR LSV curves and **(b)** *Koutecký*-*Levich* diagrams of IrFe-SACs at different rotation speeds and potentials for ORR tests


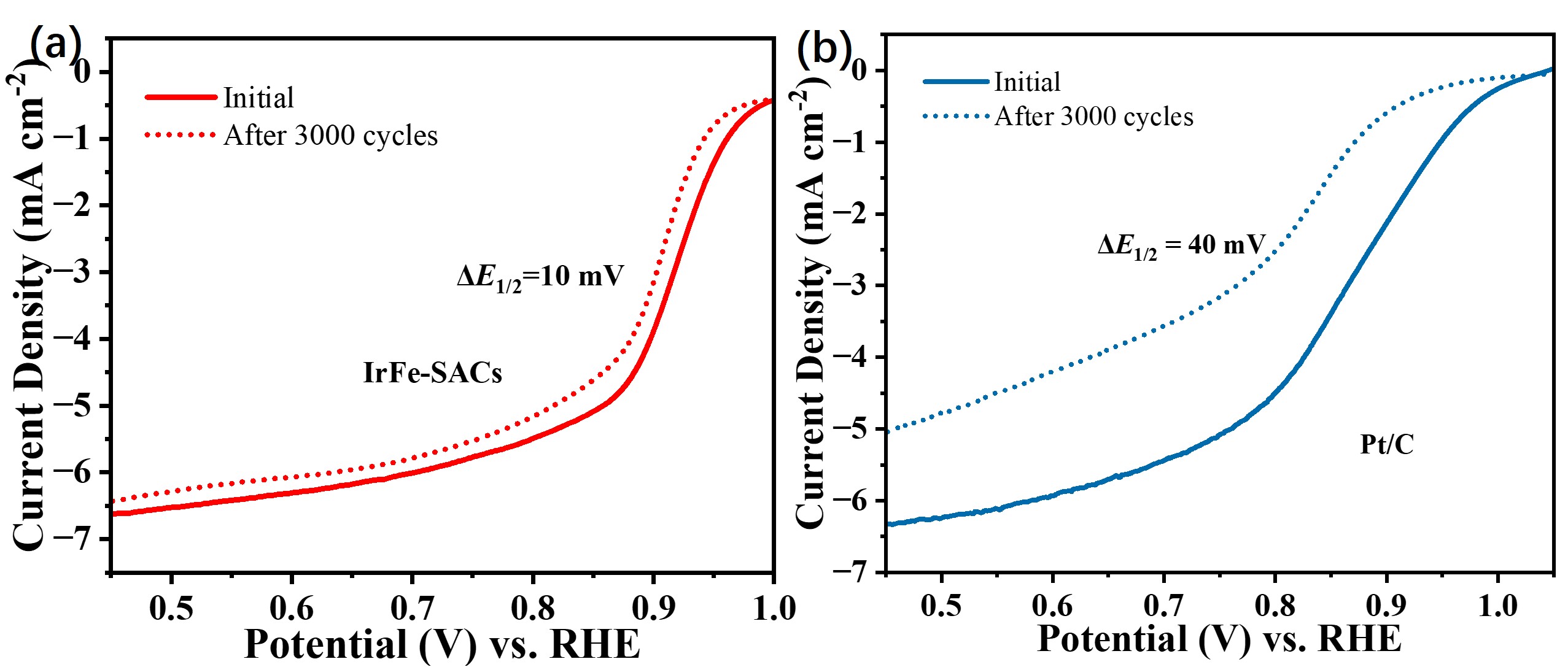


**Fig. S18** ORR polarization curves of **(a)** IrFe-SACs and **(b)** Pt/C before and after 3000 CV cycles

**
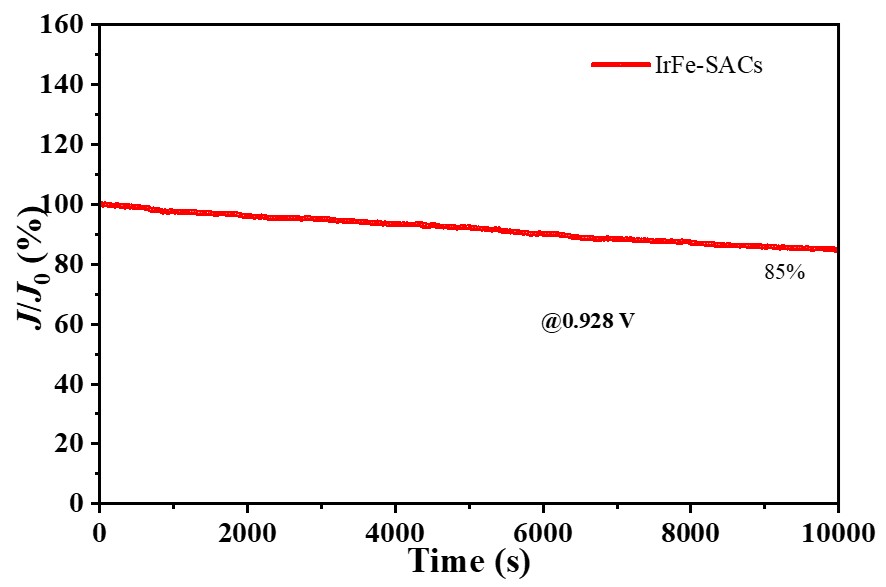
**

**Fig. S19** I-t stability measurements curve of IrFe-SACs

**Table S11** ICP results of IrFe-SACs after cycling stability tests

| **Samples** | **Mass**  **/g** | **Volume**  **/mL** | **Element** | **Readout**  **/mg L^-1^** | **Content**  **/mg kg^-1^** | **wt.%** |
| --- | --- | --- | --- | --- | --- | --- |
| **IrFe-SACs** | 0.0263 | 20 | Fe | 29.0271 | 22073.8 | 2.2074% |
|  | 0.0263 | 20 | Ir | 5.5024 | 4184.3 | 0.4184% |

**
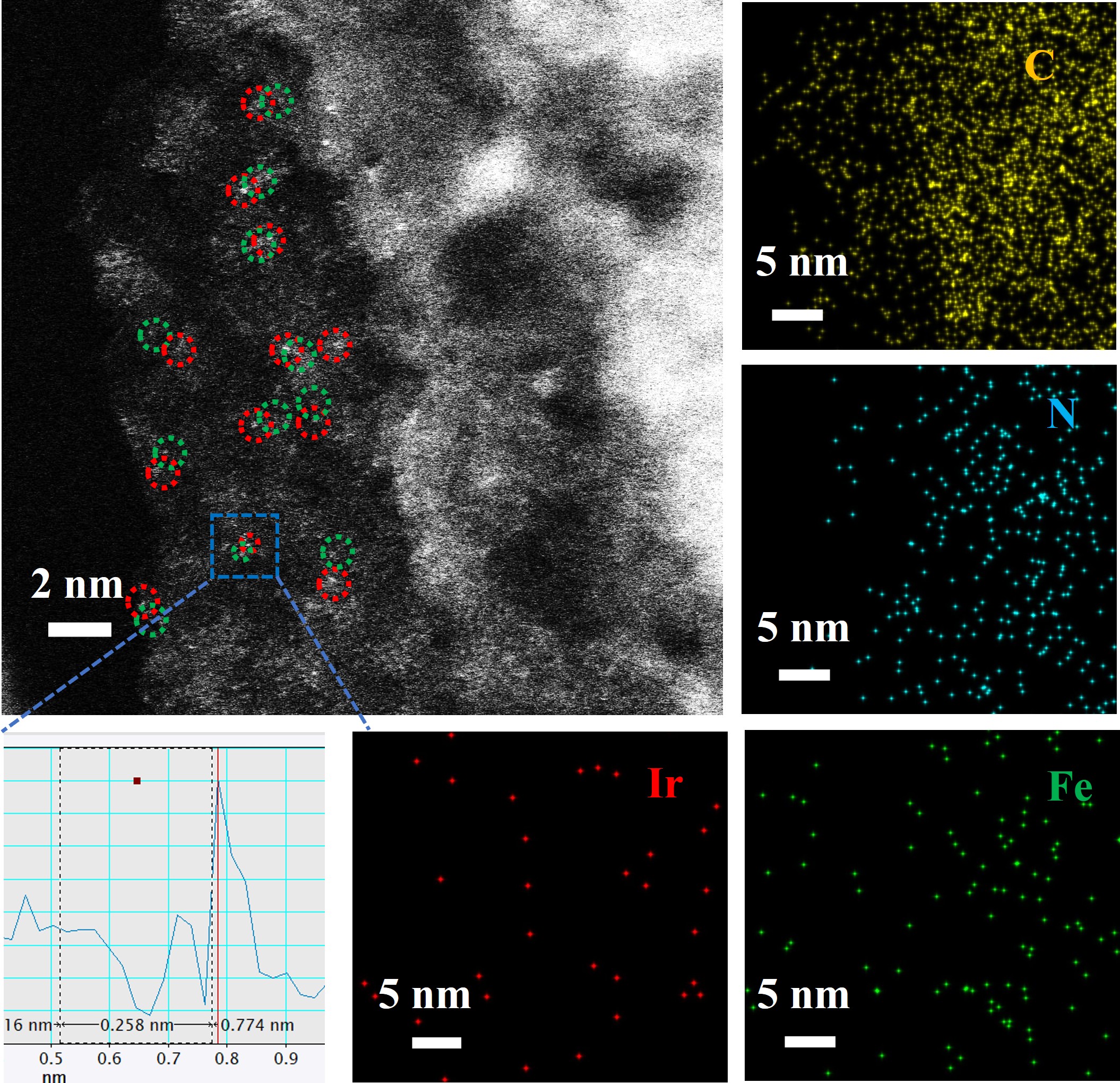
**

**Fig. S20** HAADF-STEM images, element mapping and the intensity profiles of IrFe-SACs after 3000 CV stability tests

**
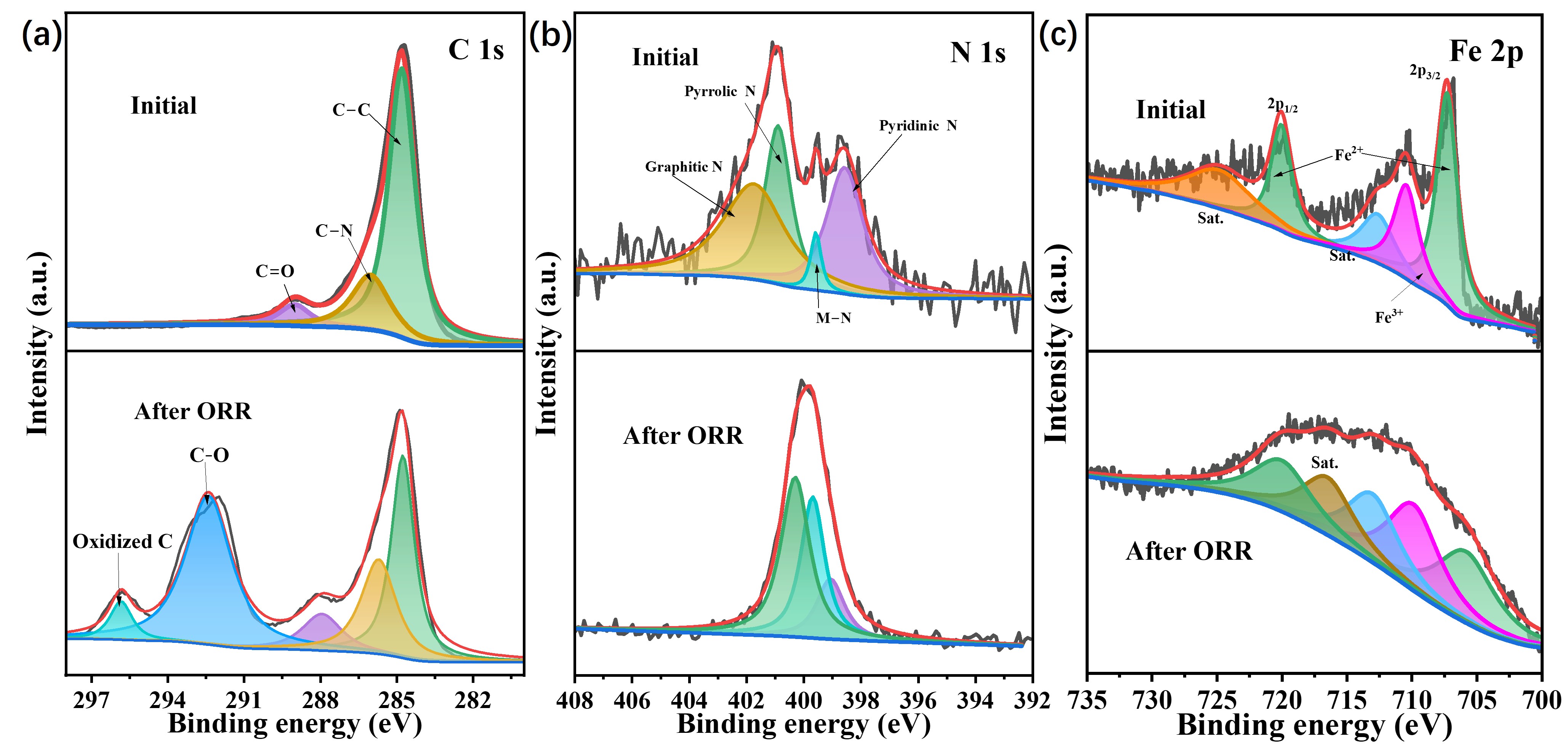
**

**Fig. S21** High-resolution scans of **(a)** C 1s, **(b)** N 1s and **(c)** Fe 2p for IrFe-SACs before and after ORR stability tests

**Fig. S22** Open circuit voltage curves of liquid ZABs based on IrFe-SACs and Pt/C-RuO_2_ air cathode

**
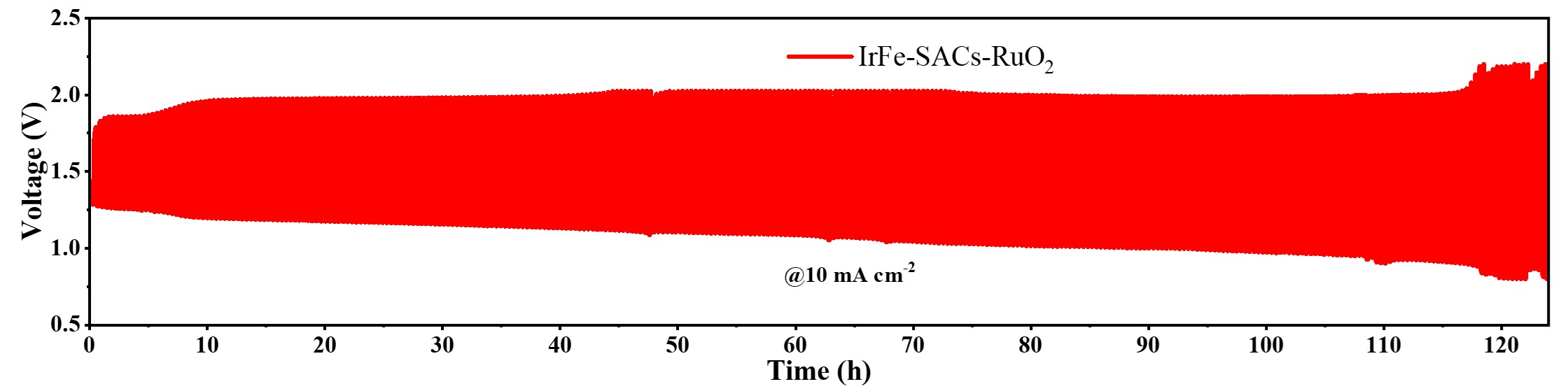
**

**Fig. S23** Galvanostatic charge-discharge cycling curves at 10 mA cm^-2^ of liquid ZABs based on IrFe-SACs-RuO_2_ air cathode

**Table S12** Performance of liquid ZABs based on recently reported oxygen electrocatalysts (-- not mentioned)

| **Catalysts** | **Power density**  **/(mW cm^-2^)** | **Specific capacity** | **Cycling stability** | **References** |
| --- | --- | --- | --- | --- |
| **IrFe-SACs** | **314** | **815.1 mAh g^-1^**  **@5 mA cm^-2^** | **20 min/cycle**  **1650 cycles**  **@5 mA cm^-2^** | **This work** |
| Fe_3_Co_7_-NC | 133 | 747 mAh g^-1^  @2 mA cm^-2^ | 20 min/cycle  600 cycles  5 mA cm^-2^ | [S11] |
| Fe-N_2_-Fe | 170 | 776.7 mAh g^-1^  @2 mA cm^-2^ | 20 min/cycle  1200 cycles  10 mA cm^-2^ | [S12] |
| FeCo-N-C-700 | 150 | 518.0 mAh g^-1^  @10 mA cm^-2^ | 10 min/cycle  252 cycles  5 mA cm^-2^ | [S13] |
| SS-Co-SAC | 195 | 714.2 mAh g^-1^  @5 mA cm^-2^ | 20 min/cycle  100 cycles  10 mA cm^-2^ | [S14] |
| Fe, Co, N−C | 198 | 726.0 mAh g^-1^  @2 mA cm^-2^ | 4 min/cycle  765 cycles  5 mA cm^-2^ | [S15] |
| CoFe−N−C | 142 | -- | 60 min/cycle  200 cycles  5 mA cm^-2^ | [S16] |
| meso/micro-FeCo-N_x_-CN-30 | 150 | -- | 20 min/cycle  120 cycles  5 mA cm^-2^ | [S17] |
| IrCo-N-C | 139 | 818.0 mAh g^-1^  @50 mA cm^-2^ | 30 min/cycle  450 cycles  5 mA cm^-2^ | [S18] |
| Ni,Fe-DSAs/NCs | 217 | 780.1 mAh g^-1^  @40 mA cm^-2^ | 240 min/cycle  125 cycles  5 mA cm^-2^ | [S19] |

**Table S13** The calculated partial atomic (Ir, Fe, O, H) mean magnetic moments of various oxygen intermediates for IrFe-SACs and Fe-SACs before and after O/OH adsorption (${\mu_{B}=e\hbar}/{2m_{e}=}$ 5.7884×10^-5^ eV T^-1^)

| **Items** | **Models** | **IrFe-SACs** | **Fe-SACs** |
| --- | --- | --- | --- |
| ***m*_O_/*****μ*_B_** | **O^*^** | -0.603 | 0.574 |
| ***m*_Ir_/*μ*_B_** |  | 0.050 | -- |
| ***m*_Fe_/*μ*_B_** |  | -1.178 | 1.229 |
| ***m*_O_/*μ*_B_** | **HO^*^** | 0.090 | 0.106 |
| ***m*_Ir_/*μ*_B_** |  | -0.047 | -- |
| ***m_Fe_*/*μ*_B_** |  | 0.929 | 1.052 |
| ***m_H_*/*μ*_B_** |  | 0.000 | 0.001 |
| ***m*_Ir_/*μ*_B_** | ***** | 0.143 | -- |
| ***m*_Fe_/*μ*_B_** |  | 1.337 | 0.000 |

**Table S14** The calculated partial atomic (Ir, Fe, O, H) mean charge of various oxygen intermediates for IrFe-SACs and Fe-SACs before and after O/OH adsorption (*e* = 1.6022×10^-19^ C)

| **Items** | **Models** | **IrFe-SACs** | **Fe-SACs** |
| --- | --- | --- | --- |
| ***Q*_O_/*e*** | **O^*^** | 4.873 | 4.872 |
| ***Q*_Ir_/*e*** |  | 8.823 | -- |
| ***Q*_Fe_/*e*** |  | 7.854 | 7.757 |
| ***Q*_O_/*e*** | **HO^*^** | 5.045 | 5.051 |
| ***Q*_Ir_/*e*** |  | 8.823 | -- |
| ***Q_Fe_*/*e*** |  | 7.604 | 7.628 |
| ***Q_H_*/*e*** |  | 0.736 | 0.740 |
| ***Q*_Ir_/*e*** | ***** | 8.859 | -- |
| ***Q*_Fe_/*e*** |  | 7.541 | 7.632 |

**
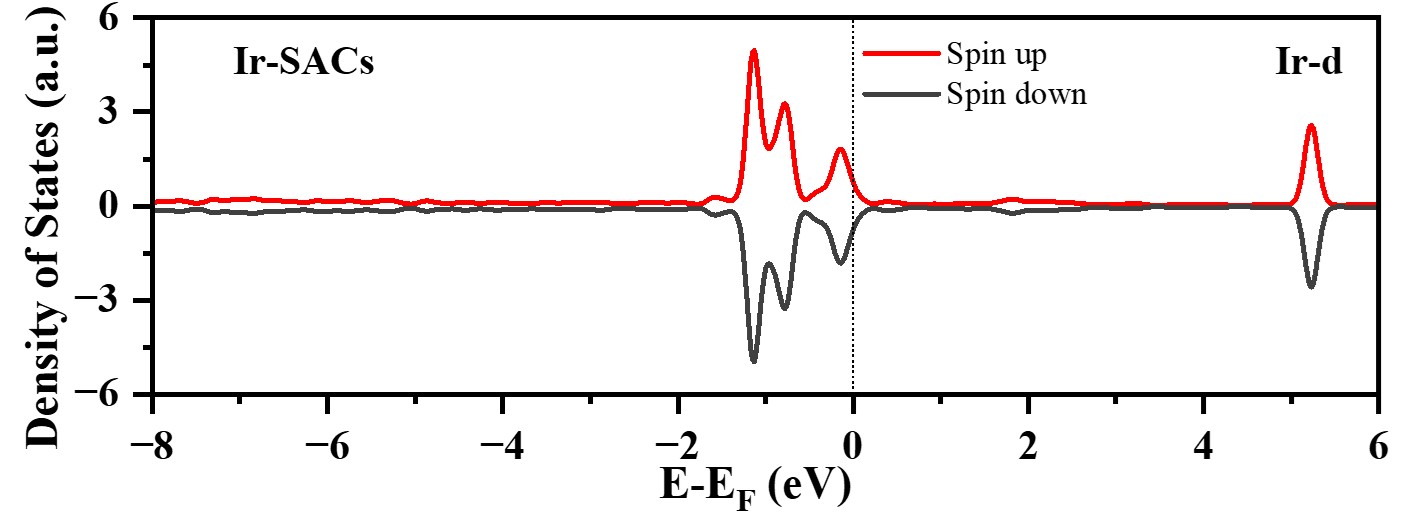
**

**Fig. S24** PDOS of Ir-d for Ir-SACs


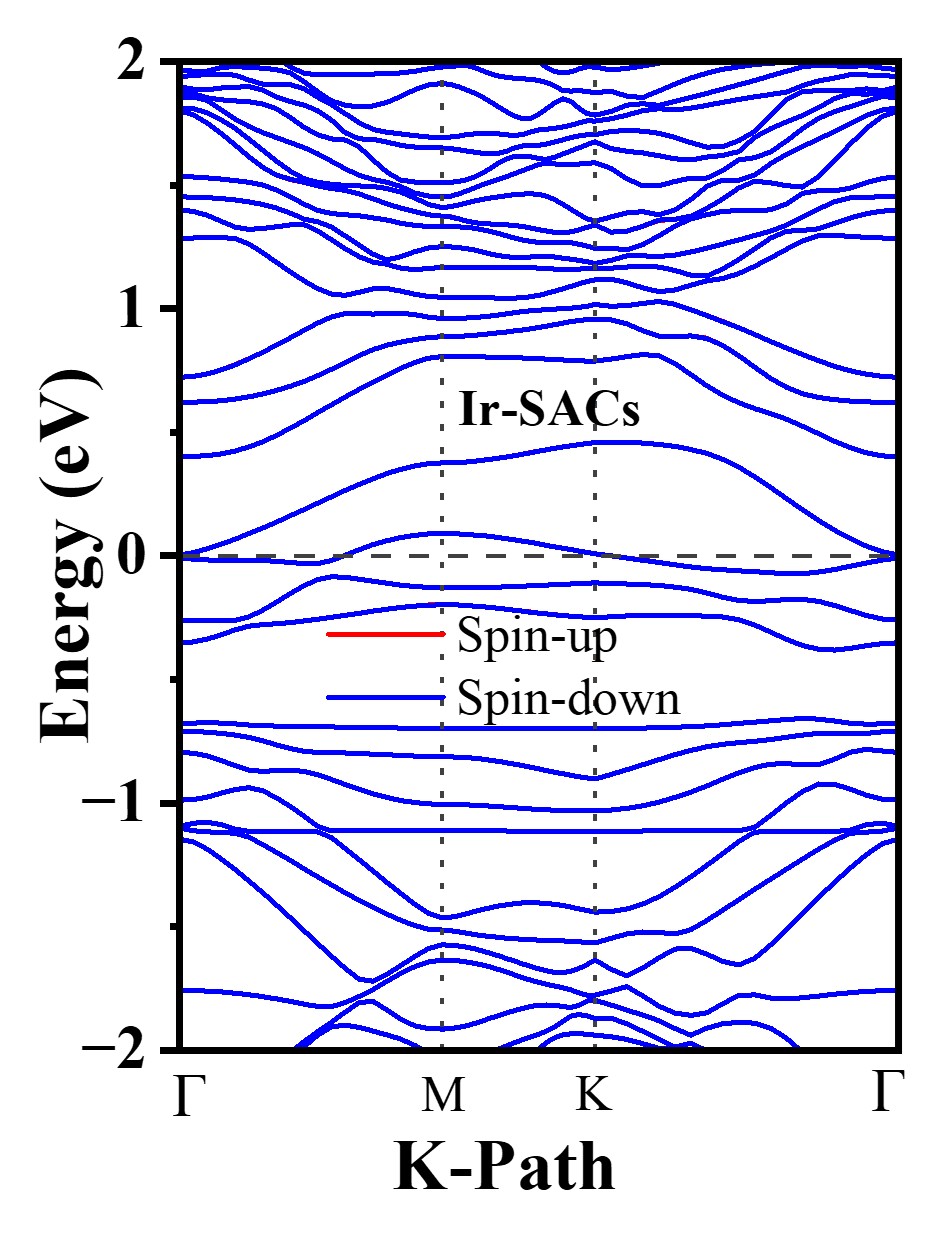


**Fig. S25** Band structure of Ir-SACs

**Table S15** The HOO(Δ*E*_1_), O (Δ*E*_2_) and HO (Δ*E*_3_) adsorption energy of Ir-SACs, Fe-SACs and IrFe-SACs

| **Models** | **Ir-SACs** | **Fe-SACs** | **IrFe-SACs** |
| --- | --- | --- | --- |
| ***E*_HOO_/eV** | -13.0239 | -13.0239 | -13.0239 |
| ***E*_*_/eV** | -1317.0225 | -1317.8284 | -1310.2555 |
| **E_HOO*_/eV** | -1332.6671 | -1332.3122 | -1324.9336 |
| ***E*_1_/eV** | -2.6207 | -1.4599 | -1.6542 |
| ***E*_O_/eV** | -1.5739 | -1.5739 | -1.5739 |
| ***E*_*_/eV** | -1317.0225 | -1317.8284 | -1310.2555 |
| ***E*_O*_/eV** | -1323.4089 | -1323.2953 | -1315.7519 |
| ***E*_2_/eV** | -4.8125 | -3.8930 | -3.9225 |
| ***E*_HO_/eV** | -7.5614 | -7.5614 | -7.5614 |
| ***E*_*_/eV** | -1317.0225 | -1317.8284 | -1310.2555 |
| ***E*_HO*_/eV** | -1328.5056 | -1327.7780 | -1320.4409 |
| ***E*_3_/eV** | -3.9217 | -2.3882 | -2.6240 |


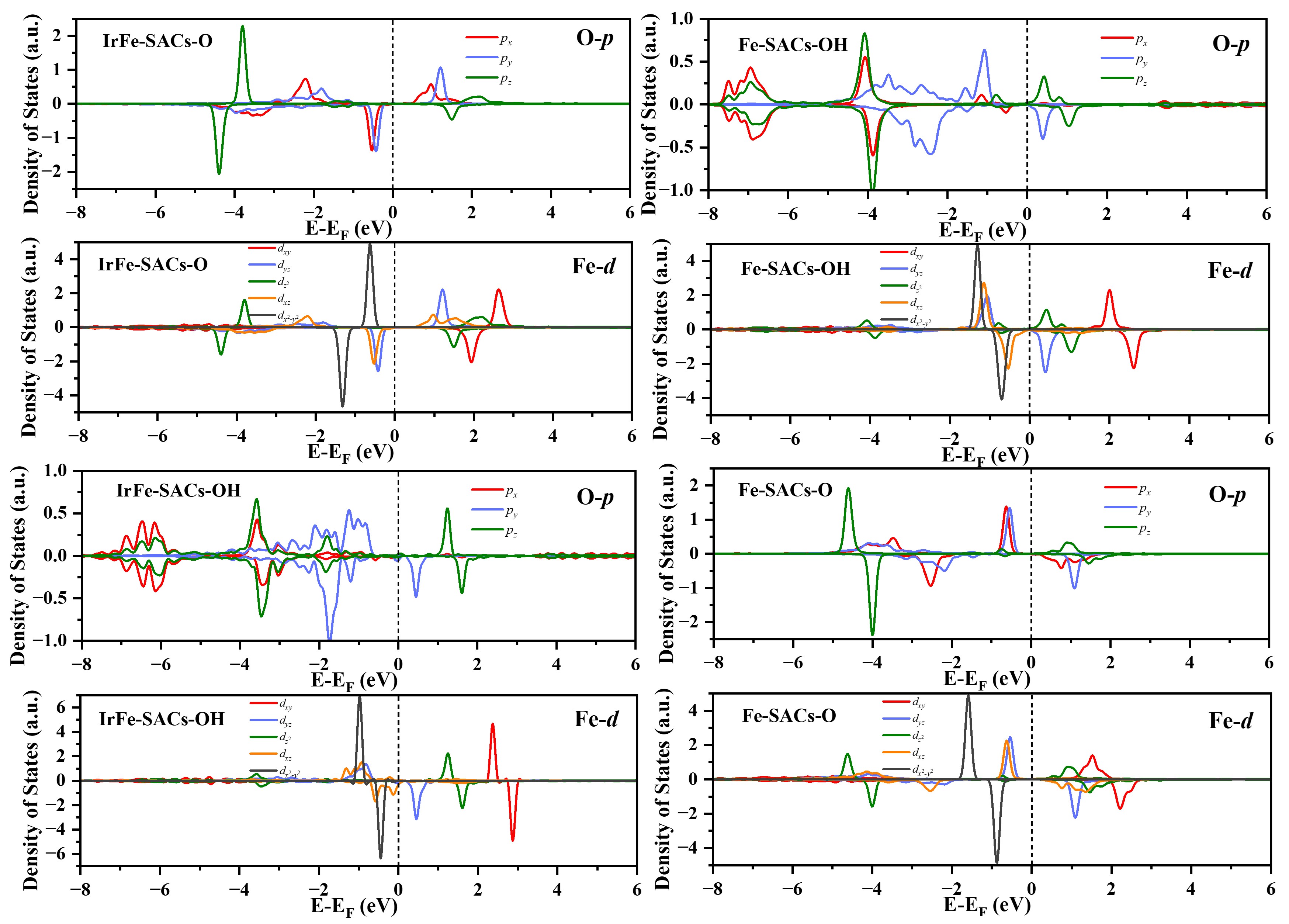


**Fig. S26** Fe-*d* and O-*p* orbital PDOS diagrams of IrFe-SACs-O/OH and Ir-SACs-O/OH


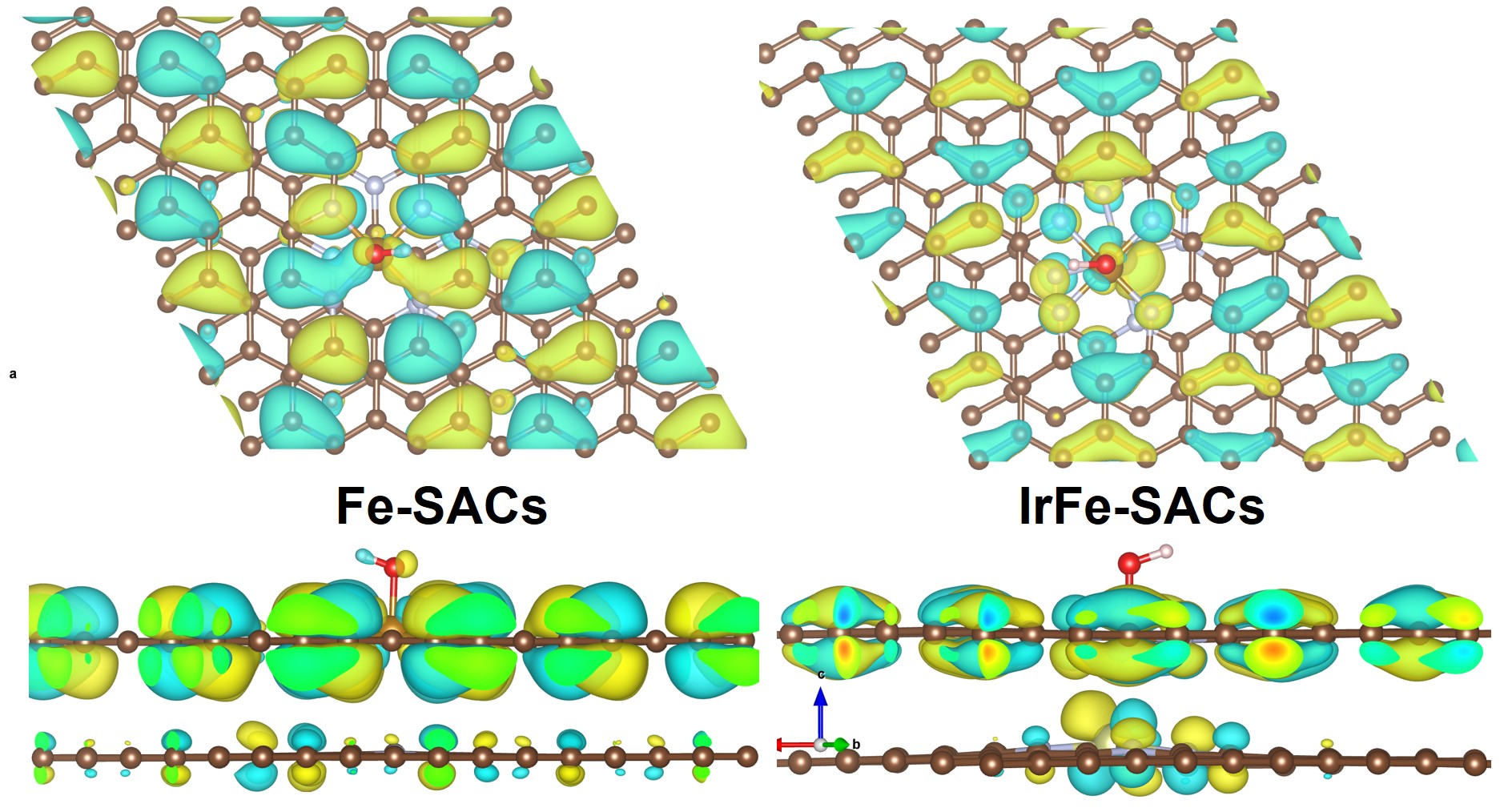


**Fig. S27** HOMO molecular orbital diagrams of Fe-SACs-OH and IrFe-SACs-OH

**Supplementary References**

1. P. Zhang, H.-C. Chen, H. Zhu, K. Chen, T. Li et al., Inter-site structural heterogeneity induction of single atom Fe catalysts for robust oxygen reduction. Nat. Commun. **15**(1), 2062 (2024). <https://doi.org/10.1038/s41467-024-46389-3>
2. B. Sun, S. Zhang, H. Yang, T. Zhang, Q. Dong et al., Revealing the active sites in atomically dispersed multi-metal–nitrogen–carbon catalysts. Adv. Funct. Mater. **34**(29), 2315862 (2024). <https://doi.org/10.1002/adfm.202315862>
3. X. Han, X. Ling, Y. Wang, T. Ma, C. Zhong et al., Generation of nanoparticle, atomic-cluster, and single-atom cobalt catalysts from zeolitic imidazole frameworks by spatial isolation and their use in zinc-air batteries. Angew. Chem. Int. Ed. **58**(16), 5359–5364 (2019). <https://doi.org/10.1002/anie.201901109>
4. L. Jiao, G. Wan, R. Zhang, H. Zhou, S.-H. Yu et al., From metal–organic frameworks to single-atom Fe implanted N-doped porous carbons: efficient oxygen reduction in both alkaline and acidic media. Angew. Chem. Int. Ed. **57**(28), 8525–8529 (2018). <https://doi.org/10.1002/anie.201803262>
5. Y. Yang, B. Li, Y. Liang, W. Ni, X. Li et al., Hetero-diatomic CoN_4_‐NiN_4_ site pairs with long-range coupling as efficient bifunctional catalyst for rechargeable Zn-air batteries. Adv. Sci. **11**(22), 2310231 (2024). <https://doi.org/10.1002/advs.202310231>
6. Q. Jing, Z. Mei, X. Sheng, X. Zou, Q. Xu et al., Tuning the bonding behavior of d-p orbitals to enhance oxygen reduction through push–pull electronic effects. Adv. Funct. Mater. **34**(3), 2307002 (2024). <https://doi.org/10.1002/adfm.202307002>
7. G. Wang, M. Zhang, G. Zhang, Z. Wang, X. Chen et al., Novel approach of diffusion-controlled sequential reduction to synthesize dual-atomic-site alloy for enhanced bifunctional electrocatalysis in acidic and alkaline media. Adv. Funct. Mater. **34**(6), 2308876 (2024). <https://doi.org/10.1002/adfm.202308876>
8. J. Qiao, Y. You, L. Kong, W. Feng, H. Zhang et al., Precisely constructing orbital-coupled Fe─Co dual-atom sites for high-energy-efficiency Zn–air/iodide hybrid batteries. Adv. Mater. **36**(32), 2405533 (2024). <https://doi.org/10.1002/adma.202405533>
9. H. Liu, L. Jiang, J. Khan, X. Wang, J. Xiao et al., Decorating single-atomic Mn sites with FeMn clusters to boost oxygen reduction reaction. Angew. Chem. Int. Ed. **62**(3), e202214988 (2023). <https://doi.org/10.1002/anie.202214988>
10. Z. Jiang, X. Liu, X.-Z. Liu, S. Huang, Y. Liu et al., Interfacial assembly of binary atomic metal-N(x) sites for high-performance energy devices. Nat. Commun. **14**(1), 1822 (2023). <https://doi.org/10.1038/s41467-023-37529-2>
11. T. Gu, D. Zhang, Y. Yang, C. Peng, D. Xue et al., Dual-sites coordination engineering of single atom catalysts for full-temperature adaptive flexible ultralong-life solid-state Zn−Air batteries. Adv. Funct. Mater. **33**(8), 2212299 (2023). <https://doi.org/10.1002/adfm.202212299>
12. S. Zhao, M. Liu, Z. Qu, Y. Yan, Z. Zhang et al., Cascade synthesis of Fe- N_2_-Fe dual-atom catalysts for superior oxygen catalysis. Angew. Chem. Int. Ed. 63(40), e202408914 (2024). <https://doi.org/10.1002/anie.202408914>
13. X. Duan, S. Ren, N. Pan, M. Zhang, H. Zheng, MOF-derived Fe, Co@N–C bifunctional oxygen electrocatalysts for Zn–air batteries. J. Mater. Chem. A **8**(18), 9355–9363 (2020). <https://doi.org/10.1039/d0ta02825h>
14. W. Xie, Y. Song, S. Li, J. Li, Y. Yang et al., Single-atomic-co electrocatalysts with self-supported architecture toward oxygen-involved reaction. Adv. Funct. Mater. **29**(50), 1906477 (2019). <https://doi.org/10.1002/adfm.201906477>
15. S. Sarkar, A. Biswas, E.E. Siddharthan, R. Thapa, R.S. Dey, Strategic modulation of target-specific isolated Fe, Co single-atom active sites for oxygen electrocatalysis impacting high power Zn-air battery. ACS Nano **16**(5), 7890–7903 (2022). <https://doi.org/10.1021/acsnano.2c00547>
16. X. Zhou, J. Gao, Y. Hu, Z. Jin, K. Hu et al., Theoretically revealed and experimentally demonstrated synergistic electronic interaction of CoFe dual-metal sites on N-doped carbon for boosting both oxygen reduction and evolution reactions. Nano Lett. **22**(8), 3392–3399 (2022). <https://doi.org/10.1021/acs.nanolett.2c00658>
17. S. Li, C. Cheng, X. Zhao, J. Schmidt, A. Thomas, Active salt/silica-templated 2D mesoporous FeCo-nx-carbon as bifunctional oxygen electrodes for zinc–air batteries. Angew. Chem. Int. Ed. **57**(7), 1856–1862 (2018). <https://doi.org/10.1002/anie.201710852>
18. M. Xiao, J. Zhu, S. Li, G. Li, W. Liu et al., 3d-orbital occupancy regulated Ir-co atomic pair toward superior bifunctional oxygen electrocatalysis. ACS Catal. **11**(14), 8837–8846 (2021). <https://doi.org/10.1021/acscatal.1c02165>
19. Z. Wang, X. Jin, R. Xu, Z. Yang, S. Ma et al., Cooperation between dual metal atoms and nanoclusters enhances activity and stability for oxygen reduction and evolution. ACS Nano **17**(9), 8622–8633 (2023). <https://doi.org/10.1021/acsnano.3c01287>
